# The Role of Glucosinolate Hydrolysis Products from *Brassica* Vegetable Consumption in Inducing Antioxidant Activity and Reducing Cancer Incidence

**DOI:** 10.3390/diseases4020022

**Published:** 2016-06-16

**Authors:** Talon M. Becker, John A. Juvik

**Affiliations:** Department of Crop Sciences, University of Illinois at Urbana-Champaign, Urbana, IL 61801-3838, USA; tbecker2@illinois.edu (T.M.B.); juvik@illinois.edu (J.A.J.)

**Keywords:** cancer, chemoprevention, glucosinolate, hydrolysis product, *Brassica*, antioxidant enzymes, quinone reductase, phase II, detoxification, Nrf2

## Abstract

The bioactivity of glucosinolates (GSs), and more specifically their hydrolysis products (GSHPs), has been well documented. These secondary metabolites evolved in the order Brassicales as plant defense compounds with proven ability to deter or impede the growth of several biotic challenges including insect infestation, fungal and bacterial infection, and competition from other plants. However, the bioactivity of GSHPs is not limited to activity that inhibits these kingdoms of life. Many of these compounds have been shown to have bioactivity in mammalian systems as well, with epidemiological links to cancer chemoprevention in humans supported by *in vitro*, *in vivo*, and small clinical studies. Although other chemopreventive mechanisms have been identified, the primary mechanism believed to be responsible for the observed chemoprevention from GSHPs is the induction of antioxidant enzymes, such as NAD(P)H quinone reductase (NQO1), heme oxygenase 1 (HO-1), glutamate-cysteine ligase catalytic subunit (GCLC), and glutathione S transferases (GSTs), through the Keap1-Nrf2-ARE signaling pathway. Induction of this pathway is generally associated with aliphatic isothiocyanate GSHPs, although some indole-derived GSHPs have also been associated with induction of one or more of these enzymes.

## 1. Introduction

**Figure 1:**
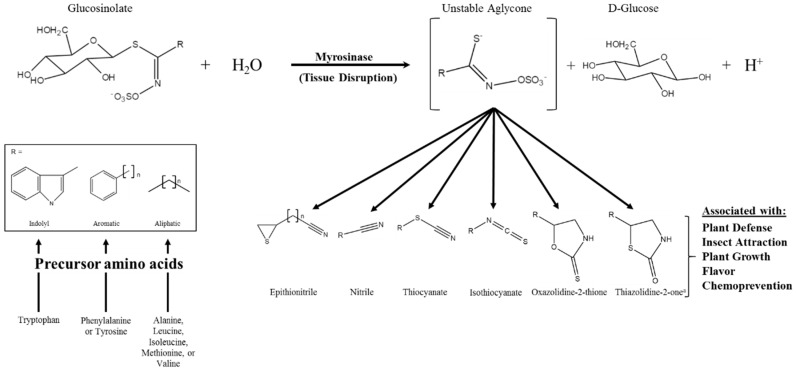
Glucosinolate hydrolysis by myrosinase, possible hydrolysis products, and the various bioactivities associated with those products; modifed from [12,19]. The generic glucosinolate side chain structures (R) can undergo further modifications at virtually every position. ^a^ Novel hydrolysis product structure recently described in [20].

Glucosinolates (GSs) are a class of amino acid-derived, sulfur-rich secondary metabolites found in the order Brassicales, which includes the scientifically and economically important genera of *Arabidopsis* and *Brassica* [1,2]. Glucosinolates are a well-defined diverse class of secondary metabolites with approximately 132 documented GSs structures by 2011 and at least a dozen additional natural structures awaiting elucidation [3,4]. Glucosinolates are generally classified based on the structure of their precursor amino acid, with aliphatic, indole, and aromatic GSs derived from methionine, tryptophan, and phenylalanine/tyrosine, respectively, being the major classes found in the *Brassica* genus [5,6]. Glucosinolates are “activated” by a class of hydrolytic enzymes called myrosinases, which, for the most part [7,8], are physically separated from GSs in intact cells [9,10,11]. Upon tissue disruption, the hydrolysis reaction mediated by myrosinase results in the formation of GS hydrolysis products (GSHPs), which are considered the bioactive component of this system (Figure 1; [12]). These compounds are well known for their role in plant defense against herbivory and pathogens [13,14,15,16]. However, perhaps more importantly to humans, they have also been associated with cancer chemoprevention [17,18].

Chemoprevention of cancer has been an active field of research since the seminal review by Wattenberg [21] in which he described early *in vivo* experiments with compounds that displayed an ability to inhibit cancer formation in animal models. To describe this observed result, he coined the term “chemoprophylaxis of carcinogenesis”, which has since evolved into “cancer chemoprevention” [22]. In the almost five decades since, the research of chemical compounds that can inhibit the formation of neoplasms and/or aid in ridding the body of neoplastic cells, through apoptosis or other means, has been steadily increasing in popularity and promise. 

Among the compounds that have shown chemopreventive qualities are the GSHPs, particularly isothiocyanates (ITCs) derived from aliphatic GS precursors [23]. From the indole GS precursor, glucobrassicin, the ITC-derived hydrolysis products indole-3-carbinol (I3C) and 3,3′-diindolylmethane (DIM) [24] have shown promising chemopreventive effects against hormone-responsive cancers, such as those of the breast, prostate, and ovaries [25]. Also, several ITCs have been linked to a number of chemopreventive mechanisms, such as: induction of cytoprotective proteins through the Keap1/Nrf2/ARE pathway, inhibition of proinflammatory responses through the regulation of the NFκB pathway, induction of cell cycle arrest and apoptosis, effects on heat shock proteins, and inhibition of angiogenesis and metastasis [26]. Inhibition of histone deacetylase (HDAC) enzymes by a number of ITCs has been implicated as at least one of the mechanisms responsible for the observed apoptotic properties of these GSHPs [26,27]. Though a significant amount of research has been done on these and other compounds from cruciferous vegetables, there is still much to be learned about the specific chemopreventive mechanisms and synergistic relationships of these compounds [28,29]. Of the several chemopreventive mechanisms of GSHPs, the best studied is probably their action on the Keap1/Nrf2/ARE (antioxidant response element) pathway, which is discussed in more detail below as it is a central mechanism by which GSHPs reduce oxidative stress in human cells.

Other compounds known to affect cellular oxidative stress are the vitamins A, C, and E, which are also relatively abundant in *Brassica* vegetables. An excellent review by Bodupalli *et al.* [30] discusses how these known antioxidant compounds may contribute to reduction of oxidative stress in humans synergistically with ITCs. This is proposed to be achieved through the induction of ARE-dependent enzymes by GSHPs that maintain the free radical scavenging capabilities of the vitamins, increasing their biologically effective life span.

## 2. The Influence of Diet on Cancer Development

The twentieth century saw many great medical revolutions, from penicillin to the polio vaccine, which led to a severe decline in the number and proportion of total deaths due to infectious disease in the U.S. However, as infectious diseases were better managed, death due to chronic health problems, such as heart disease and cancer, became more common. In 1900, cancer and heart disease reportedly caused 64 and 137.4 of every 100,000 deaths, respectively. By 2010, those numbers had increased to 185.9 for cancer and 192.9 for heart disease, making these the most common causes of death among Americans as a whole [31]. The increased prevalence of these diseases has led to increased research into their causes and modes of prevention. Prevention and treatment of heart disease has experienced great strides, as mortality from heart disease has been on a steady decline since the 1970s [32]. In fact, cancer has surpassed heart disease as the leading cause of death in individuals between the ages of 40 and 79 [33]. For this reason, cancer is now a major focus of medical research. Specifically, understanding the mechanisms of carcinogenesis, metastasis, and the overall progression of cancer is of the utmost importance in accomplishing the goal of decreasing new cancer incidence. It is known that there are certain genetic factors that can lead to increased risk for several types of cancer. However, the effect of these genetic defects can only be attributed to a relatively minor proportion of total cancer incidence for most types of neoplasms [34]. Whereas, diet has been estimated to be associated with 20%–42% of cancer cases [35]. Many studies have supported this conclusion, showing positive associations between cancer rates and certain foods such as meat (red meat in particular), saturated fats, trans-fats, and eggs [36,37]. However, not all foods have a positive association with cancer risk. Certain components of an individual’s diet, such as consumption of vegetables, fruits, cereals, and olive oil as well as a high ratio of monounsaturated to saturated lipids, can have a negative association with cancer risk [37]. In other words, there is evidence that certain foods and dietary choices can affect an individual’s risk for cancer (reviewed by Erdman Jr. *et al.* [38]).

The link between certain dietary components and lower cancer risk helps to corroborate the results of early research indicating that people who live in the Mediterranean, and more specifically, adhere to the classic Mediterranean diet, have a lower cancer incidence [37,39,40]. Although there are many characteristics of the Mediterranean diet that have been shown to influence cancer rates, consumption of vegetables from the *Brassica* genus may be one of the most important contributing factors [41]. The cancer-preventative qualities associated with *Brassica* vegetables could be partially linked to the high levels of vitamins A, C, E, and other antioxidants found in these vegetables [30]. However, the general scientific consensus due to a number of epidemiological, *in vitro*, and *in vivo* studies is that the chemopreventive effects of *Brassica* vegetable consumption are in large part due to the presence of GSs, and more specifically, their GSHPs [42,43].

## 3. Glucosinolate Hydrolysis Products and Their Chemopreventive Bioactivity

Chemopreventive bioactivity often refers to the ability of a chemical compound, or mixture of compounds, to induce phase I (PI)/phase II (PII) detoxification enzymes and/or antioxidant enzymes in the human body. Antioxidant (AO) enzymes generally act in the regulation of glutathione metabolism and quenching of free radicals via one- and two-electron reductions, thereby contributing to the reduction of oxidative stress. AO enzymes include catalases (CAT), superoxide dismutases (SOD), glutathione reductases (GSR), glutathione peroxidases (GPX), glutaredoxins (GLRX), thioredoxins (TXN), thioredoxin reductases (TXNRD), heme-oxygenase 1 (HO-1), and NAD(P)H:quinone oxidoreductase 1 (NQO1) [30,44]. Phase I/phase II detoxification enzymes are often called biotransformation enzymes because, as a team, they transform toxic xenobiotics into non-toxic forms that can be excreted from the body. In this process, PI enzymes perform various reactions that alter the lipophilic xenobiotic target compound in a way that allows it to react with PII enzymes [45]. Phase II enzymes can directly act on some xenobiotics as well as perform conjugation reactions on the PI products. Most PII enzymes are transferases: UDP-glucuronosyltransferases (UGTs), sulfotransferases (SULTs), glutathione S-transferases (GSTs), N-acetyltransferases (NATs), and S- and O-methyltransferases (MTs) [30]. NQO1 is often included in the list of PII enzymes [44], although it is also often referred to it as an AO enzyme. The product of the PII reaction is a more polar compound that can be readily excreted by the body either through passive or active transport. In general, PII/AO enzymes maintain a balanced redox state in mammalian cells by controlling levels of reactive oxygen species (ROS), which is done by maintaining glutathione (GSH) and thioredoxin levels as well as helping to maintain the equilibrium between NAD^+^/NADH and NADP^+^/NADPH [46]. Despite their role in detoxification, PI enzymes can also activate compounds known as procarcinogens [30]. When a procarcinogen reacts with a PI enzyme, such as one of many cytochrome P450s (CYPs), the non-toxic procarcinogen is transformed into a toxic, carcinogenic substance. For this reason, the induction of PI enzymes by a given compound may decrease the overall chemopreventive effect of that compound. On the other hand, PII enzymes typically perform conjugation reactions that result in a compound that is more polar, and subsequently less toxic, than the non-conjugated form [30,47,48,49]. For this reason, compounds that induce only PII enzymes are considered more beneficial, as there is little chance of negative effects. Compounds with this quality are known as monofunctional inducers, as opposed to bifunctional inducers that induce both PI and II enzyme activity. Sulforaphane (SF), the hydrolysis product of the GS called glucoraphanin, has been shown to be a PII monofunctional inducer [50]. In fact, ITCs like SF are generally considered to be PII monofunctional inducers [51]. Therefore, future prospects for *Brassica* breeders could be to increase SF or other ITC levels in their crops with little fear of affecting the safety of the crop’s consumption.

However, this may not be true for all GSs and GSHPs. While there is no strong evidence of negative effects from GS consumption in humans, this has been observed quite frequently in livestock that eat *Brassica* forages as a major part of their diet. The adverse symptoms seen in livestock from the consumption of high levels of GSs/GSHPs are often attributed to the consumption of goitrin, an oxazolidine-2-thione GSHP formed from the spontaneous cyclization of the ITC product of progoitrin hydrolysis [52]. Although, goitrin may not be the only GSHP that causes negative health effects upon ingestion of high doses. Research has shown teratogenic effects of other GSHPs in murine models, most notably allyl ITC (AITC) from sinigrin, 1-cyano-2-hydroxy-3,4-epithiobutane (CETB) from progoitrin, and iberin from glucoiberin [53]. The common symptoms seen in livestock often attributed to the overconsumption of GS/GSHPs are: slowed growth [54], impaired movement and general disorientation [55], impaired fertility [56], and damage to the thyroid, gastro-intestinal tract, and/or liver [57,58]. Because of what has been seen in animals, caution must be taken when manipulating GS and GSHP profiles. Although it seems that some of the most bioactive GSHPs show beneficial health effects at the normally consumed dosages, breeders and food scientists should take care to screen for any negative health effects from GS and GSHP profile manipulation of agricultural products.

Although there have been negative effects observed in livestock linked to high levels of GS and/or GSHP consumption, several GSHPs have displayed significant chemopreventive activity in a number of *in vitro* studies. Common *Brassica* ITCs, such as SF and phenethyl ITC (PEITC), have proven to either inhibit carcinogenesis or induce cancer cell growth arrest and apoptosis in several cell types including: breast [59], bladder [60], colon [61], ovary [62], blood [63], skin [64], and prostate cells [65]. The mechanism by which these compounds accomplish this task is not fully understood and probably not universal, but some of the known effects of ITC treatment on cell metabolism include modulation of gene expression and alternative gene splicing [66]. Perhaps most importantly, several ITCs have been shown to increase the activity of nuclear factor (erythroid-derived 2)-like 2, also known as NFE2L2 or Nrf2 [67]. When activated, Nrf2 increases transcription rates of a number of AO and PII genes, ultimately leading to cells that are less likely to develop neoplasia [68,69]. While it appears that most ITCs from *Brassica* or related vegetables induce PII enzymes, there may be differences between aromatic, indole, and aliphatic ITCs in their modulation of PI enzyme activity [70,71,72]. A summary of experiments utilizing ITCs, ITC-derived compounds, and GSHP mixtures as treatments testing their effect on PII/AO gene transcript/protein abundance and/or enzyme activity can be found in Table 1.

In addition to the direct benefits of Nrf2 induction on cellular xenobiotic metabolism, there is also evidence of possible crosstalk between the Nrf2 and NFκB pathways (reviewed by Li *et al.* [106]) as well as evidence that these compounds block the phosphorylation and subsequent degradation of the protein that acts to sequester NFκB in the cytosol. Degradation of this protein would lead to increased nuclear translocation of NFκB, which is associated with inducing transcription of pro-inflammatory genes commonly found to be upregulated in cancer cells (reviewed by Cheung and Kong [107]). This is just one example of multiple chemopreventive bioactivities of GSHPs, which demonstrates the complexity underlying the crosstalk and co-regulation that exists between cellular stress mechanisms and their effect on the initiation, promotion, and/or progression of cancer. 

## 4. Nrf2/Keap1/ARE Signaling Cascade

Regarding chemopreventive bioactivity induced by GSHPs, one mechanism that has been well described is the Keap-1/Nrf2/ARE signaling cascade present in mammalian cells (reviewed by Jaramillo and Zhang [108] and Kansanen *et al.* [109]). In this signaling pathway, expression of many PII and AO enzymes is promoted by the binding of Nrf2 transcription factors to ARE sequences in the promoter regions of these genes [110]. It is believed that under basal conditions, the transcription factor Nrf2 is sequestered by Keap1 in the cytoplasm. The generally accepted mechanism by which this occurs is that two Keap1 proteins, which are part of a larger Keap1-Cul3-E3 ubiquitin ligase complex, bind to the ETGE and DLG motifs in the Neh2 domain of Nrf2 and promote polyubiquitination and subsequent proteasomal degradation of Nrf2 [69,111,112,113,114]. However, when Keap1 reacts with any of a number of bioactive molecules, polyubiquitination may be impeded. 

It is hypothesized that the cysteine residues of Keap1 can react with a number of electrophilic compounds. The modification of the thiol groups of these cysteine residues is thought to alter the conformation of Keap1 [115,116,117]. This, in turn, causes the Keap1 dimer to release the DLG motif of Nrf2, which is suggested to prevent Nrf2 polyubiquitination and degradation [111,112,113]. This proposed mechanism results in Keap1 becoming saturated with Nrf2, allowing newly synthesized Nrf2 to be freely translocated to the nucleus. Once in the nucleus, Nrf2 forms a heterodimer with one of a number of small masculoaponeurotic fibrosarcoma (sMaf) proteins [118] allowing for the binding of the heterodimer to antioxidant response elements (AREs) in the promoter regions of a number of genes involved in cell metabolism and detoxification [118,119,120], while other Maf proteins can act as repressors [121]. In the absence of Nrf2, sMaf proteins form homodimers and bind to AREs, but effectively act as repressors due to their inability to stimulate transcriptional activation [122]. This mechanism for Nrf2/Keap1 regulation of ARE-dependent genes, including those encoding PII and AO enzymes, has been well supported by a number of other studies not already mentioned [123,124,125]. The Nrf2/Keap1/ARE signaling cascade is discussed in more detail by a number of reviews [30,126,127,128].

## 5. *In Vitro* Evidence of ARE-Dependent Gene Induction by ITCs

The utility of ITCs for inducing ARE-dependent gene transcription and activity has been thoroughly studied for several years. Popular approaches for such research have been reviewed by Fuentes *et al.* [129]. There have been a number of ITCs found in *Brassica* crops that have shown the ability to induce PII and/or AO gene expression/activity, although perhaps through slightly different mechanisms. These include but are not limited to SF, PEITC, AITC, benzyl ITC (BITC), iberin, erucin, and the ITC-derived compounds, I3C and DIM. These GSHPs can be found in a number of *Brassica* crops, although with variable abundance due to environmental and genetic factors. A few of the *Brassica* crops considered to be good sources of one or several of these GSHPs are broccoli, various mustards, cabbage (most types), and gai-lan (Chinese broccoli) (Becker *et al.*, in preparation for submission [130]).

SF induces ARE-dependent gene expression through reaction with Keap1. The reaction of SF with Keap1 cysteine residues (primarily C38, C151, C368, and C489; [131]) results in the formation of thionoacyl adducts. However, unlike some other inducers of Nrf2-dependent genes, SF was shown not to result in polyubiquinaton of Keap1 [132,133]. It is believed that the formation of thionoacyl adducts on Keap1 in the presence of SF reduces the binding affinity of Keap1 for Cul3, resulting in an inability to eliminate Nrf2 through proteasomal degradation and migration of free Nrf2 to the nucleus [131,134,135]. Additionally, there is some evidence that a transcriptional coregulator called SPBP may be involved in SF perception and Nrf2 induction [136]. The effects of SF treatment on AO and PII enzymes *in vitro* have been studied in a wide variety of human and murine cell lines, including LNCaP, PC-3, TSU-Pr1, MDA PCa 2a, MDA PCa 2b, MDA-MB-231, transgenic adenocarcinoma of mouse prostate (TRAMP) C1, HeLa, HT-29, CaCo-2, HepG2, Hepa1c1c7 and MCF-7 [85,102,137,138,139]. The protective effect of SF against oxidative stress has been well documented as this compound was identified several years ago as a strong inducer of PII and AO enzymes, and subsequently, an effective chemopreventive agent. 

Much of the early work with SF was reviewed by Fahey and Talalay [140], with a more recent review conducted by Guerrero-Beltran *et al.* [141]. The studies reviewed by these authors generally attributed the protective effect of SF, no matter the tissue, to increases in transcription and/or activity of one or several PII and/or AO enzymes. Some studies also went as far as to show these effects to be Nrf2-dependent using Nrf2 inhibitors or Nrf2-deficient cell lines. Other studies not included in the review by Guerrero-Beltran *et al.* have shown similar results. For example, Mizuno *et al.* [76] showed SF treatment (1 µM) to increase nuclear translocation of Nrf2 as well as expression of γ-glutamylcysteine synthetase (γ-GCS) and HO-1 in rat neuronal cells. In this experiment, the researchers hypothesized that the protective effect of SF observed is primarily due to the increase in γ-GCS expression and subsequent increase in intercellular reduced GSH, based on results from experiments with inhibitors of γ-GCS and HO-1. Also, as a side note, the level of SF used in this and many SF bioactivity studies is well within the range of 0.943–2.27 µM reported to be present in human plasma and erythrocytes 1 h following ingestion of approximately 200 µmol of ITCs in the form of broccoli sprouts [142]. 

While results from several studies using different tissues show similar results from SF treatment, there is evidence that there are mechanisms other than the Keap1 pathway by which SF induces Nrf2 expression/activity. This is shown in a study using mouse TRAMP cells, a prostate cancer model that was previously reported as containing an epigenetic mechanism that leads to decreased Nrf2 and downstream gene expression [143]. Using these cells, Zhang *et al.* [102] reports that SF treatment results in the demethylation of the first five CpG sites in the Nrf2 promoter, leading to an increase in Nrf2 mRNA and protein expression. The beneficial effect of SF on oxidative stress may not be limited to induction of Nrf2-dependent genes. In addition to the well-demonstrated effectiveness of SF in that capacity, there is evidence that SF may act directly as a radical scavenger from superoxide through the action of SOD and from hydrogen peroxide without enzymatic interaction [144]. 

The mechanism for PEITC induction of ARE-dependent genes is believed to occur via increased extracellular signal-regulated kinase (ERK)- and/or c-Jun-NH_2_-kinase (JNK)-dependent phosphorylation of Nrf2. Upon treatment with PEITC, increased phosphorylation by ERK and/or JNK results in improved migration of Nrf2 to the nucleus and ARE-dependent gene expression. Support for this mechanism comes from results that show an attenuation of the effects of PEITC treatment by ERK/JNK inhibition and or genetic knockout [145,146]. This mechanism also appears to be partially responsible for the Nrf2 induction by SF and AITC [146]. In a recent study, PEITC-induced Nrf2-dependent PII and AO enzyme induction was tested in HepG2 cells. At concentrations of 1 µM and 5 µM, PEITC was shown to significantly increase Nrf2 mRNA, nuclear Nrf2 protein, and phosphorylated nuclear Nrf2 by approximately 2-fold, 1.3-fold, and 1.5-fold, respectively. Both PII and AO enzymes showed increases in expression and protein abundance following PEITC treatment. Both tested concentrations of PEITC increased expression and protein abundance of all four major classes of GSTs while NQO1 expression and abundance was only significantly increased by 5 µM PEITC treatment. Several AO enzymes (SOD, CAT, GPX, and GSR) also responded to both tested concentrations of PEITC with significant increases in both transcript and protein abundance [44]. In another study, PEITC was also shown to increase expression of HO-1 and an ARE-driven luciferase reporter gene in PC-3 cells. Even though no statistical analysis was presented in this study, a clear positive trend was observed in both HO-1 and ARE/luciferase up to concentrations of 7.5–10 µM PEITC. However, ARE/luciferase activity decreased at 20 µM, and there were signs of cytotoxicity with long term exposure to higher concentrations [147]. 

In addition to SF and PEITC, other ITCs common in *Brassica* vegetables have shown evidence of the ability to induce the ARE-dependent genes. For example, SF, PEITC, BITC, and AITC were generally shown to increase transcript and protein abundance of γ-GCS, HO-1, and NQO1 when applied to NIH3T3 fibroblast cells at the lowest dose tested (5 µM) [74]. A similar study using the same four ITCs and mouse skin papilloma cells found analogous results, with significant increases in NQO1 and GST activity. The authors also showed that the measured area under time-concentration curve of intracellular concentrations of a given ITC were strongly correlated with the induction of the measured enzymes as well as glutathione (GSH) content. Comparable correlations were found for all four ITCs using human HepG2 cells and an ARE-luciferase reporter [148]. While Nrf2 signaling is no doubt one of the major contributors to the chemopreventive bioactivity of PEITC, several other mechanisms besides Nrf2 activation have been proposed to contribute, including induction of apoptosis and cell cycle arrest. Several of these potential mechanisms are discussed in depth in a review by Qin *et al.* [149]. 

A few studies also exist exploring the effect of I3C treatment on AO and PII enzymes *in vitro*. One such study was briefly discussed above for its evaluation of PEITC induction of AO and PII enzymes. I3C showed similar results to PEITC in this study, although the tested concentrations were double that of PEITC (2 and 10 µM). Even though I3C was shown to significantly increase transcript and protein abundance of many of the same PII and AO enzymes as PEITC, some differences existed. In general, PEITC treatments resulted in greater increases in transcript and protein for the genes tested, even at lower concentrations. These differences were most extreme for transcript abundance of a number of genes (SOD, GPX, and all tested GSTs), but were less evident when evaluating the corresponding protein abundance [44]. In yet another study, TRAMP C1 cells modified to contain an ARE-luciferase reporter were treated with I3C at concentrations of 25, 50, 75, and 100 µM. Treatments were shown to increase luciferase reporter activity in a dose-dependent manner, with an effective dose (statistically different from control) of 50 µM. Likewise, Nrf2 and several downstream genes (glutamate-cysteine ligase catalytic subunit [GCLC], NQO1, and HO-1) were upregulated, in terms of mRNA levels. Induction was, again, dose-dependent with effective doses being reached at 75, 25, 25, and 75 µM for Nrf2, GCLC, NQO1, and HO-1, respectively [77]. The mechanism(s) for I3C induction of Nrf2 have yet to be fully elucidated, but one study suggests that at least one mechanism is indirect, acting through suppression of the production of reactive oxygen species (ROS) upon treatment of the cytotoxic compounds, such as dexamethasone (Dex). In this study, I3C treatments as low as 5 µM resulted in a negation (statistically speaking) of cytotoxic effects of Dex as determined by cell viability comparisons to a control in MC3T3-E1 osteoblastic cells. Also, 10 µM and 20 µM I3C pretreatment prior to treatment with Dex reduced the production of superoxide by 30% and 40%, respectively, when measured as percent of the control. Superoxide levels were also found, roughly, to negatively associate with Nrf2 and downstream gene expression in the Dex and Dex+I3C treatments. This led the authors to hypothesize that in their study, the induction of Nrf2 and downstream genes by I3C was largely due to the attenuation of Dex-induced ROS production and any Nrf2-repression mechanisms that those ROS levels may have imposed [150]. 

While the experiments discussed above use I3C as a treatment, there is evidence that I3C undergoes oligomerization in cell culture [151], just as it does in acidic conditions like the stomach [152]. This means that other compounds, such as 3,3′-diindolylmethane (DIM), may be responsible for some of the bioactivity associated with I3C. Yet, a reminder of just how complex the mechanisms of bioactivity underlying the chemopreventive properties of these compounds can be seen in an experiment done by Szaefer *et al.* [94]. In this study, three different breast cell lines, two of which were tumorigenic, were treated with both I3C (10, 30, and 50 µM) and DIM (5 and 10 µM). Levels of transcript and protein were measured for a number of genes, including NQO1 and GST-π (GSTP). I3C and DIM showed similar patterns of induction for these genes, but some slight differences are seen depending on the gene being measured and the cell line, an indication of possible interactions between treatment and genotype. However, it is difficult to determine if the observed differences are real, as no ANOVA was reported for this study to test for statistical significance of any of these effects. One thing that is suggested from the results of the Szaefer *et al.* study is that the effective dose of DIM for induction of Nrf2 and downstream genes is much lower than I3C, as treatments of 5 µM and 10 µM DIM showed similar levels of induction of both mRNA and protein of NQO1 and GSTP as 30 µM and 50 µM I3C, respectively. Along with Nrf2 induction, I3C and its derivatives exhibit chemopreventive bioactivity through a number of other mechanisms, including suppression of proliferation of cancer cells from various tissues through regulatory repression of cyclin-dependent kinases, induction of apoptosis, and modulation of expression of several transcription factors (reviewed by Aggarwal and Ichikawa [153]). However, these indole-derived GSHPs are generally considered to be inferior to the aliphatic-derived ITCs in terms of chemopreventive potential, mainly due to their bifunctional nature [70,71]. 

## 6. Response of AO and PII Enzymes to ITC Treatment in Animal Studies

As with *in vitro* studies, SF has been the most studied ITC in animal models. SF has generally been shown to inhibit PI enzymes while inducing PII enzymes, such as NQO1, TXNRD, GST, and HO-1, in a variety of tissues/cell types [23,73]. However, the inhibitory effect of SF on PI enzymes may not be universal for all genetic backgrounds. A report by Hu *et al.* [81] detected increases in PI gene expression following treatment with SF in Nrf2 wild-type mice compared to Nrf2 knockouts. This inconsistency in results from different studies concerning the action of SF on PI enzymes is indicative that the mechanisms of bioactivity of SF, probably the most-studied ITC, are still not fully understood. 

One factor that has been considered as research on SF and other GSHPs moved from *in vitro* to *in vivo* studies is that the bioactivity of a given GSHP depends on the level of bioavailability, or ease of absorption by the body or a given tissue, of that compound [154]. Studies show high bioavailability of SF in CD-1 mice following oral administration of 2.5 mg/kg broccoli sprout preparations. Modeling of SF uptake in this experiment indicated that SF absorption is principally perfusion-limited [155]. Similarly high levels of bioavailability have been measured in humans [156,157,158], independent of GSTP1 polymorphism [156]. It has also been observed that the bioavailability of SF is greatly dependent upon whether or not functional myrosinase is included with SF’s precursor GS, glucoraphanin. Use of fresh *Brassica* tissue and/or supplementation with myrosinase results in a higher bioavailability of SF in both mice [155] and humans [156,157,158]. 

The AO and PII inductive effects of PEITC have also been tested in animal models. One such study treated Nrf2 wild-type and knockout mice with a dosage of 40 mg/kg PEITC. Microarray analysis of liver mRNA revealed upregulation for a large number of genes both 3 h and 12 h after treatment. These included a number of AO and PII genes along with several genes associated with heat shock proteins, ubiquitin/26 S proteasome subunits, and lipid metabolism [88]. Like SF, PEITC appears to be highly bioavailable. Oral bioavailability in rats was calculated to be at or near 100% for doses of 10 and 100 μmol/kg [159]. In addition, half-life of PEITC in rat is reported to be between 4 and 22 h, depending on the tissue [160]. The high bioavailability of PEITC, much like SF, contributes to the promise of this phytochemical being absorbed in effective doses through dietary means. A disadvantage of PEITC compared to SF is that this compound is much more volatile [161], less water soluble [162], and therefore more likely to be lost from the *Brassica* vegetable during preparation (*i.e.*, after cutting of plant tissue leads to hydrolysis of gluconasturtiin, the precursor GS of PEITC). However, consumption of salad crops like watercress, from a genus closely related to *Brassica*, can deliver large doses of gluconasturtiin, active myrosinase, and subsequently, PEITC at estimated levels of 37 µmol per ounce [163]. Another aliphatic ITC common to *Brassica* crops that is gaining attention is erucin, from the precursor GS glucoerucin. This ITC was recently found to induce HO-1 expression *in vitro* and *in vivo*, possibly acting through a similar mechanism of Nrf2 activation as PEITC [96]. Interestingly, erucin can also undergo interconversion to SF *in vivo*, although the interconversion ratio of SF and erucin is not consistent across individuals [156].

As mentioned above, indole-derived GSHPs are considered less effective chemopreventive compounds due to their bifunctional induction capabilities. Both I3C and DIM have been tested for their high dose toxicity and PI enzyme induction in Sprague-Dawley rats using doses equivalent to 5–7x the maximal human dose for I3C and 1 or 10x for DIM. After 12 months of feeding, no major indicators of toxicity were observed, but the I3C diet was shown to significantly increase PI protein levels. The high dosage DIM diet also caused PI protein induction, but generally at a lower level than I3C and with some differences between tissues for both compounds [70]. These results along with those of previously discussed studies indicate the superiority of DIM as a chemopreventive agent compared to I3C due to its lower bifunctional induction [70] and lower effective dose for the induction of PII enzymes *in vitro* [94]. In some trials, DIM treatments showed results similar to those of *in vitro* studies. In one such study, DIM administered intravenously to male Sprague-Dawley rats at a dose of 10 mg/kg resulted in significant increases in mRNA levels of NQO1, GSTP1, and UGT1A1 in blood lymphocytes, peaking approximately 1–2 h after DIM treatment. Concentrations of DIM in plasma were also measured, but due to the poor bioavailability of DIM, calculated in this study as 1%–3%, plasma concentrations were relatively low, peaking around 9 µg/mL five minutes after administration. DIM was also shown to be quickly metabolized, with a terminal half-life of 0.737 h [164]. The low bioavailability of DIM has been corroborated by other research [165,166]. This fact has raised concerns that the levels of DIM needed to elicit a response in mice may not be achievable through dietary intake of *Brassica* crops. Wu *et al.* [164] calculated that the dosage used in their experiment would translate to a human dosage of 1.6 mg/kg. Concentrations of DIM in both green and red kale leaf tissue, a crop containing relatively high amounts of DIM’s precursor GS (glucobrassicin), have been shown to be less than 1 µg/mg dried tissue [167]. One caveat is that liver concentrations of DIM have been shown to be six to eight times that of plasma in mice [166], indicating that a smaller dose may be necessary to reach effective dose levels in liver cells [164].

## 7. Clinical and Epidemiological Evidence for the Importance of ITCs in Chemoprevention

Although there has been significant evidence that a diet rich in GSHPs can have chemopreventive effects, this relationship often is not found to be significant in epidemiological studies. A review by Higdon *et al.* [42] discusses this common observation. The authors looked at several epidemiological studies for the four types of cancer with the highest mortality rates in the U.S.: lung, colorectal, breast, and prostate. Though there are obvious differences between these cancers, there seemed to be some commonalities in the results of the different epidemiological studies. For each type of cancer, the authors concluded that even though many small case-control studies found a significant inverse relationship between cruciferous vegetable intake and cancer rates, the larger prospective cohort studies often did not find the same significant relationship. There were a few exceptions to this generalization. Some of the prospective cohort studies found significant inverse relationships, but usually in specific populations within the full data set of the study [168,169,170,171,172]. The different results between the two types of studies may be due to participation bias in case-control studies, wherein control groups who choose to participate are more health conscious and have better eating habits compared to non-participating controls [173]. However, as the authors of the review indicate, the inconsistency of results may be due to a more complex relationship between cruciferous vegetable intake and cancer risk than what was previously thought.

Although results of different epidemiological studies can often be contradictory, a review of 87 case-control studies that were performed prior to 1996 indicated that a majority (67%) show an inverse relationship between cancer risk and cruciferous vegetable consumption [174]. Building on this, Jeffery and Keck [175] make the case that there is enough evidence to proceed with larger clinical trials testing the efficacy of purified SF, semi-purified SF, and/or whole broccoli for inducing detoxification enzyme activity. The authors reviewed a number of epidemiological, *in vitro*, and *in vivo* studies examining the link between cruciferous vegetable intake, or bioactive GSHPs, and cancer. In this article, all epidemiological studies reviewed showed a significant (*p* < 0.05) decrease in cancer risk associated with cruciferous vegetable consumption for various types of cancer including: bladder [176], lung [177], lymphoma [178], prostate [179,180], breast [181], kidney [182], and ovarian [183]. These results were further supported with a number of *in vitro* studies showing several anti-tumorigenic activities in various mammalian cancer cell types after treatment with SF. Also reported were several *in vivo* animal model studies showing decreased tumor growth, incidence, and/or multiplicity using broccoli, SF, and ITCs as treatments. Finally, several small clinical studies were reported that further corroborated the observed results in epidemiological, *in vitro*, and animal model studies described above. These studies reported positive associations between broccoli or broccoli sprout consumption and several different biomarkers linked to efficacy of the treatment for inducing cellular detoxification mechanisms. More recent reviews and/or meta-analyses have also corroborated the conclusion that consumption of cruciferous (*Brassica*) vegetables, in general, results in a decreased risk of several cancer types [184,185,186,187,188,189,190,191].

Singh and Singh [23] expanded on the case made by Jeffery and Keck, widening the scope to include PEITC and BITC. Again, the authors cited evidence supporting the chemopreventive potential of these compounds through a number of mechanisms, including cellular ROS modulation, and called for a transition into large-scale clinical research of dietary ITCs. However, the authors of both reviews discussed above admitted to challenges that exist when considering the transition into human clinical trials with ITCs, including formulation of ITC treatments that are repeatable and suitable for oral administration as well as identifying proper dosage and treatment scheduling due to the rapid metabolism and excretion of ITC derivatives/conjugates.

Another major factor to be considered when planning clinical trials testing the chemopreventive properties of ITCs, possibly through the use of dietary intervention with *Brassica* vegetables, is the inter- and intra-individual variation in bioavailability of SF, and likely other GSHPs. Although the mechanism of ITC uptake is likely complex, bioavailability of these compounds is generally attributed to differences in the GST genotype and/or microbiota composition of the subject [158,192,193]. Yet, the magnitude of the impact of those factors on SF and other GSHP bioavailability/bioactivity is still not fully clear. It is hypothesized that due to the major role of GSH in the conjugation and elimination of SF, GSTM1-null individuals would benefit more from SF consumption due to the increased time that SF would be in the body [42,194]; however, this has not been fully confirmed by epidemiological evidence [73]. A number of studies have shown no significant effect of GST genotype on ITC uptake and/or Nrf2 and downstream gene activation [156,195], while other research shows a significant genotype effect [196,197,198]. The effect of GST genotype may depend on the specific GST being considered as well as the level of dietary ITC consumption [197]. The true influence of microbiota on GSHP bioavailability has also been questioned, as germ-free mice show only slightly altered profiles of excreted GS metabolites [199]. Also, even though high and low ITC excretors have been described [200], specific gut microorganisms could not be associated with this capability [201].

There is still work to be done towards implementing large-scale fully randomized placebo control trials (reviewed by Fahey *et al.* [202]). However, several smaller clinical trials have occurred over the past couple decades, generally using *Brassica* vegetables as the ITC delivery matrix. Several of these studies were reviewed by Boddupalli *et al.* [30] and James *et al.* [73]. In general, the research discussed in these reviews showed induction of ARE-mediated enzymes and/or reduction of biomarkers for oxidative stress accompanying treatment with GSHPs. One study in particular showed that ingestion of a broccoli soup made with broccoli containing three times the normal glucoraphanin resulted in increased SF uptake in a dose-dependent manner [80]. This result indicates that improvement of *Brassica* crops for increased content of certain favorable GSs would be worthwhile and would result in higher intake of dietary ITCs for those who consumed said crops. A series of studies by Riso *et al.* not included in the aforementioned reviews reported similar beneficial results following dietary intervention with broccoli. Specifically, two of these studies reported a reduction of oxidized purines in smokers and a reduction in H_2_O_2_-induced strand breaks following broccoli consumption [203,204]. Also, this group reported an increase in GST activity only for GSTM1-positive individuals 6 h following broccoli consumption compared to 3 h and 24 h [205].

## 8. Conclusions

The results of these reports discussed above suggest that the level of the chemopreventive effect of *Brassica* vegetables may depend on the interaction of several variables, including the level of consumption of other dietary factors (vitamins, lipids, other phytochemicals, *etc.*) [30,206] and the individual’s genotype/metabolism [177,196,207,208]. In addition, the GS and subsequent GSHP profiles (presence/absence of certain GS/GSHPs) [42], as well as the presence/absence of active myrosinase [156,157,158], are also important variables in determining the chemopreventive effect of consuming a given *Brassica* vegetable. The activation of Nrf2 and downstream genes by *Brassica* ITCs and ITC-derived carbinols is a major factor in the chemopreventive properties displayed by these compounds. However, it is not the only chemopreventive mechanism stimulated by these compounds (e.g., apoptosis, cell cycle arrest, epigenetic regulation).

While there is a bulk of *in vitro* and *in vivo* evidence that many aliphatic-derived ITCs and perhaps some indole-derived GSHPs can be delivered at effective levels through modest increases in dietary intake of *Brassica* vegetables containing these compounds, caution has still been advised by the academic community when considering how large the ITC doses used in human trials can safely be. In high concentrations, these compounds have been shown to have genotoxic potential [209] and lead to cellular stress through alkylation and depletion of cellular thiols, damage to mitochondria, and elevated levels of reactive oxygen species (reviewed by Zhang *et al.* [210]). Also, there have been a few reports of increased cancer incidence in some animal models following ITC treatment, possibly depending on the specific ITC and timing of ITC treatment (pre- or post-initiation) [23]. Furthermore, constitutive activation of Nrf2 in cancer cells may be associated with drug resistance (reviewed by Huang *et al.* [211]). Despite these possible complications, the case has been made, partly in the two reviews by Jeffery and Keck [175] and Singh and Singh [23], for progression into larger-scale human clinical trials testing the chemopreventive and post-initiation cancer suppression potential of dietary ITCs. While many of the early studies show promising results in the modulation of Nrf2 and other chemopreventive molecular mechanisms with GSHPs, there is still much to learn about how these phytochemicals alter cellular metabolism in humans. Specifically, understanding the reasons behind inter- and intra-individual variation for ITC uptake, the effect of ITC mixture and/or delivery matrix, and the correct treatment dosage/timing for desired metabolic response are major goals of ongoing and future research, including human clinical trials.

## Figures and Tables

**Table 1:** Summary of human, animal, and *in vitro* studies of the effects of glucosinolate hydrolysis products on phase II and antioxidant enzymes; updated from [73].

PII/AO Enzyme	GSHP	Dosage/Treatment	Type of Study; System Used	Fold Change ^a^	Type of Change	Reference
CAT	I3C	2 µM	*in vitro*; HepG2 cells	1.2	Expression	Krajka-Kuźniak *et al.* (2015) [44]
CAT	I3C	10 µM	*in vitro*; HepG2 cells	1.7	Expression	Krajka-Kuźniak *et al.* (2015) [44]
CAT	I3C	2 µM	*in vitro*; HepG2 cells	1.2	Protein	Krajka-Kuźniak *et al.* (2015) [44]
CAT	I3C	10 µM	*in vitro*; HepG2 cells	1.3	Protein	Krajka-Kuźniak *et al.* (2015) [44]
CAT	PEITC	1 µM	*in vitro*; HepG2 cells	1.5	Expression	Krajka-Kuźniak *et al.* (2015) [44]
CAT	PEITC	5 µM	*in vitro*; HepG2 cells	1.6	Expression	Krajka-Kuźniak *et al.* (2015) [44]
CAT	PEITC	1 µM	*in vitro*; HepG2 cells	1.3	Protein	Krajka-Kuźniak *et al.* (2015) [44]
CAT	PEITC	5 µM	*in vitro*; HepG2 cells	1.3	Protein	Krajka-Kuźniak *et al.* (2015) [44]
GCL	AITC	5 µM	*in vitro*; NIH3T3 cells	2.9	Expression	Ernst *et al.* (2011) [74]
GCL	AITC	10 µM	*in vitro*; NIH3T3 cells	4	Expression	Ernst *et al.* (2011) [74]
GCL	AITC	25 µM	*in vitro*; NIH3T3 cells	5	Expression	Ernst *et al.* (2011) [74]
GCL	BITC	5 µM	*in vitro*; NIH3T3 cells	1.5	Expression	Ernst *et al.* (2011) [74]
GCL	BITC	10 µM	*in vitro*; NIH3T3 cells	2	Expression	Ernst *et al.* (2011) [74]
GCL	BITC	25 µM	*in vitro*; NIH3T3 cells	3.2	Expression	Ernst *et al.* (2011) [74]
GCL	DIM	5 µM	*in vitro*; NIH3T3 cells	1.6	Expression	Ernst *et al.* (2011) [75]
GCL	DIM	10 µM	*in vitro*; NIH3T3 cells	1.9	Expression	Ernst *et al.* (2011) [75]
GCL	DIM	25 µM	*in vitro*; NIH3T3 cells	2.4	Expression	Ernst *et al.* (2011) [75]
GCL	PEITC	5 µM	*in vitro*; NIH3T3 cells	3.8	Expression	Ernst *et al.* (2011) [74]
GCL	SF	5 µM	*in vitro*; NIH3T3 cells	2.7	Expression	Ernst *et al.* (2011) [75]
GCL	SF	5 µM	*in vitro*; NIH3T3 cells	2.7	Expression	Ernst *et al.* (2011) [74]
GCL	SF	10 µM	*in vitro*; NIH3T3 cells	2.8	Expression	Ernst *et al.* (2011) [75]
GCL	SF	10 µM	*in vitro*; NIH3T3 cells	2.9	Expression	Ernst *et al.* (2011) [74]
GCL	SF	0.1 µM	*in vitro*; fetal Wistar rats; primary striatal neuronal cultures	1.4	Protein	Mizuno *et al.* (2011) [76]
GCL	SF	1 µM	*in vitro*; fetal Wistar rats; primary striatal neuronal cultures	2.1	Protein	Mizuno *et al.* (2011) [76]
GCL	SF	1 µM; 6 h treatment	*in vitro*; fetal Wistar rats; primary striatal neuronal cultures	1.6	Protein	Mizuno *et al.* (2011) [76]
GCL	SF	1 µM; 12 h treatment	*in vitro*; fetal Wistar rats; primary striatal neuronal cultures	3.1	Protein	Mizuno *et al.* (2011) [76]
GCL	SF	1 µM; 24 h treatment	*in vitro*; fetal Wistar rats; primary striatal neuronal cultures	1.5	Protein	Mizuno *et al.* (2011) [76]
GCL	SF	1 µM; 36 h treatment	*in vitro*; fetal Wistar rats; primary striatal neuronal cultures	0.6	Protein	Mizuno *et al.* (2011) [76]
GCL	SF	10 µM	*in vitro*; fetal Wistar rats; primary striatal neuronal cultures	1.7	Protein	Mizuno *et al.* (2011) [76]
GCLC	I3C	25 µM	*in vitro*; TRAMP C1 cells	3	Expression	Wu *et al.* (2012) [77]
GCLC	I3C	50 µM	*in vitro*; TRAMP C1 cells	3.4	Expression	Wu *et al.* (2012) [77]
GCLC	I3C	75 µM	*in vitro*; TRAMP C1 cells	5.2	Expression	Wu *et al.* (2012) [77]
GCLC	MIX ^b^	15% broccoli seed/85% RM1 feed for 7 d	*in vivo*; Nrf2(+/+) mice; stomach and small intestine	5	Protein	McWalter *et al.* (2004) [78]
GCLC	SF	9 µmol per day	*in vivo*; ICR mice; small intestine cells	4	Expression	Thimmulappa *et al.* (2002) [79]
GCLM	SF	344 µmol single dose	Human clinical; gastric mucosa	3 (2.5) ^c^	Expression	Gasper *et al.* (2007) [80]
GPX	I3C	2 µM	*in vitro*; HepG2 cells	1.1	Expression	Krajka-Kuźniak *et al.* (2015) [44]
GPX	I3C	2 µM	*in vitro*; HepG2 cells	1.1	Protein	Krajka-Kuźniak *et al.* (2015) [44]
GPX	I3C	10 µM	*in vitro*; HepG2 cells	1.3	Expression	Krajka-Kuźniak *et al.* (2015) [44]
GPX	I3C	10 µM	*in vitro*; HepG2 cells	1.2	Protein	Krajka-Kuźniak *et al.* (2015) [44]
GPX	PEITC	1 µM	*in vitro*; HepG2 cells	2.2	Expression	Krajka-Kuźniak *et al.* (2015) [44]
GPX	PEITC	1 µM	*in vitro*; HepG2 cells	1.3	Protein	Krajka-Kuźniak *et al.* (2015) [44]
GPX	PEITC	5 µM	*in vitro*; HepG2 cells	3.4	Expression	Krajka-Kuźniak *et al.* (2015) [44]
GPX	PEITC	5 µM	*in vitro*; HepG2 cells	1.3	Protein	Krajka-Kuźniak *et al.* (2015) [44]
GPX3	SF	90 mg/kg; 12 h after treatment	*in vivo*; Nrf2(+/+) mice; liver	2.1	Expression	Hu *et al.* (2006) [81]
GSR	I3C	2 µM	*in vitro*; HepG2 cells	1.9	Expression	Krajka-Kuźniak *et al.* (2015) [44]
GSR	I3C	2 µM	*in vitro*; HepG2 cells	1.3	Protein	Krajka-Kuźniak *et al.* (2015) [44]
GSR	I3C	10 µM	*in vitro*; HepG2 cells	2.3	Expression	Krajka-Kuźniak *et al.* (2015) [44]
GSR	I3C	10 µM	*in vitro*; HepG2 cells	1.4	Protein	Krajka-Kuźniak *et al.* (2015) [44]
GSR	PEITC	1 µM	*in vitro*; HepG2 cells	1.7	Expression	Krajka-Kuźniak *et al.* (2015) [44]
GSR	PEITC	1 µM	*in vitro*; HepG2 cells	1.4	Protein	Krajka-Kuźniak *et al.* (2015) [44]
GSR	PEITC	5 µM	*in vitro*; HepG2 cells	2.3	Expression	Krajka-Kuźniak *et al.* (2015) [44]
GSR	PEITC	5 µM	*in vitro*; HepG2 cells	1.5	Protein	Krajka-Kuźniak *et al.* (2015) [44]
GSR	SF	5 µM	*in vitro*; rat cardiomyocytes	1.9–2.1 ^d^	Activity	Angeloni *et al.* (2009) [82]
GSR	SF	5 µM	*in vitro*; rat cardiomyocytes	1.5–2 ^d^	Expression	Angeloni *et al.* (2009) [82]
GSR	SF	5 µM	*in vitro*; rat cardiomyocytes	1.2–1.5 ^d^	Protein	Angeloni *et al.* (2009) [82]
GSR	SF	90 mg/kg; 12 h after treatment	*in vivo*; Nrf2(+/+) mice; liver	2	Expression	Hu *et al.* (2006) [81]
GSR	SF	0.1 µM	*in vitro*; mouse cortical neurons	2	Activity	Vauzour *et al.* (2010) [83]
GSTA	I3C	2 µM	*in vitro*; HepG2 cells	1.9	Expression	Krajka-Kuźniak *et al.* (2015) [44]
GSTA	I3C	2 µM	*in vitro*; HepG2 cells	1.2	Protein	Krajka-Kuźniak *et al.* (2015) [44]
GSTA	I3C	10 µM	*in vitro*; HepG2 cells	1.8	Expression	Krajka-Kuźniak *et al.* (2015) [44]
GSTA	I3C	10 µM	*in vitro*; HepG2 cells	1.3	Protein	Krajka-Kuźniak *et al.* (2015) [44]
GSTA	PEITC	1 µM	*in vitro*; HepG2 cells	3.6	Expression	Krajka-Kuźniak *et al.* (2015) [44]
GSTA	PEITC	1 µM	*in vitro*; HepG2 cells	1.3	Protein	Krajka-Kuźniak *et al.* (2015) [44]
GSTA	PEITC	5 µM	*in vitro*; HepG2 cells	5.2	Expression	Krajka-Kuźniak *et al.* (2015) [44]
GSTA	PEITC	5 µM	*in vitro*; HepG2 cells	1.6	Protein	Krajka-Kuźniak *et al.* (2015) [44]
GSTA	SF	0.2 µM	*in vitro*; rat hepatic Clone 9 cells	3.1	Protein	Lii *et al.* (2010) [84]
GSTA	SF	1 µM	*in vitro*; rat hepatic Clone 9 cells	4.5	Protein	Lii *et al.* (2010) [84]
GSTA	SF	5 µM	*in vitro*; rat hepatic Clone 9 cells	6.9	Protein	Lii *et al.* (2010) [84]
GSTA1	SF	5 µM	*in vitro*; rat cardiomyocytes	1.5–2 ^d^	Expression	Angeloni *et al.* (2009) [82]
GSTA1	SF	10 µM	*in vitro*; human prostatic cancer cells (LNCaP)	1.7	Expression	Brooks *et al.* (2001) [85]
GSTA1	SF	10 µM	*in vitro*; human prostatic cancer cells (MDA Pca 2A)	1.7	Expression	Brooks *et al.* (2001) [85]
GSTA1	SF	10 µM	*in vitro*; human prostatic cancer cells (MDA Pca 2B)	1.4	Expression	Brooks *et al.* (2001) [85]
GSTA1	SF	10 µM	*in vitro*; human prostatic cancer cells (PC3)	1	Expression	Brooks *et al.* (2001) [85]
GSTA1	SF	10 µM	*in vitro*; human prostatic cancer cells (TSU-Pr1)	1	Expression	Brooks *et al.* (2001) [85]
GSTA1	SF	11 µM in onion/broccoli extract	Human clinical; enterocytes	2	Expression	Petri *et al.* (2003) [86]
GSTA1	SF	10 µM	*in vitro*; human Caco-2 cells	3	Expression	Petri *et al.* (2003) [86]
GSTA1	SF	11 µM in onion/broccoli extract	*in vitro*; human Caco-2 cells	1.7	Expression	Petri *et al.* (2003) [86]
GSTA1/2	I3C	0.5% (*w*/*w*) supplemented RM1 feed	*in vivo*; Nrf2(+/+) mice; intestinal cytosol	2.3	Protein	McMahon *et al.* (2001) [87]
GSTA1/2	SF	3 µmol/g supplemented RM1 feed	*in vivo*; Nrf2(+/+) mice; intestinal cytosol	1.4	Protein	McMahon *et al.* (2001) [87]
GSTA2	PEITC	40 mg/kg; 3 h after treatment	*in vivo*; Nrf2(+/+) mice; liver	2.6	Expression	Hu *et al.* (2006) [88]
GSTA2	SF	90 mg/kg; 3 h after treatment	*in vivo*; Nrf2(+/+) mice; liver	2.8	Expression	Hu *et al.* (2006) [81]
GSTA2	SF	90 mg/kg; 12 h after treatment	*in vivo*; Nrf2(+/+) mice; liver	4.4	Expression	Hu *et al.* (2006) [81]
GSTA3	I3C	0.5% (*w*/*w*) supplemented RM1 feed	*in vivo*; Nrf2(+/+) mice; intestinal cytosol	2.2	Protein	McMahon *et al.* (2001) [87]
GSTA3	SF	3 µmol/g supplemented RM1 feed	*in vivo*; Nrf2(+/+) mice; intestinal cytosol	1.8	Protein	McMahon *et al.* (2001) [87]
GSTA4	I3C	0.5% (*w*/*w*) supplemented RM1 feed	*in vivo*; Nrf2(+/+) mice; intestinal cytosol	3.8	Protein	McMahon *et al.* (2001) [87]
GSTA4	SF	90 mg/kg; 3 h after treatment	*in vivo*; Nrf2(+/+) mice; liver	2.1 (2) ^c^	Expression	Hu *et al.* (2006) [81]
GSTA4	SF	90 mg/kg; 12 h after treatment	*in vivo*; Nrf2(+/+) mice; liver	2.5 (2.7) ^c^	Expression	Hu *et al.* (2006) [81]
GSTA4	SF	3 µmol/g supplemented RM1 feed	*in vivo*; Nrf2(+/+) mice; intestinal cytosol	1.9	Protein	McMahon *et al.* (2001) [87]
GST ^e^	AITC	40 µmol per kg body wt. for 5 d	*in vivo*; Sprague-Dawley rats; bladder	1.9	Activity	Munday and Munday (2004) [ 89]
GST ^e^	AITC	40 µmol per kg body wt. for 5 d	*in vivo*; Sprague-Dawley rats; duodenum	1.1	Activity	Munday and Munday (2004) [ 89]
GST^e^	AITC	40 µmol per kg body wt. for 5 d	*in vivo*; Sprague-Dawley rats; forestomach	1.4	Activity	Munday and Munday (2004) [ 89]
GST ^e^	Erucin	40 µmol per kg body wt. for 5 d	*in vivo*; Sprague-Dawley rats; bladder	1.7	Activity	Munday and Munday (2004) [ 89]
GST ^e^	Erucin	40 µmol per kg body wt. for 5 d	*in vivo*; Sprague-Dawley rats; duodenum	1.1	Activity	Munday and Munday (2004) [ 89]
GST ^e^	Erucin	40 µmol per kg body wt. for 5 d	*in vivo*; Sprague-Dawley rats; forestomach	1.3	Activity	Munday and Munday (2004) [ 89]
GST ^e^	I3C	0.5% (*w*/*w*) supplemented RM1 feed	*in vivo*; Nrf2(+/+) mice; intestinal cytosol	1.3	Activity	McMahon *et al.* (2001) [87]
GST ^e^	Iberin	40 µmol per kg body wt. for 5 d	*in vivo*; Sprague-Dawley rats; bladder	2.0	Activity	Munday and Munday (2004) [ 89]
GST ^e^	Iberin	40 µmol per·kg body wt. for 5 d	*in vivo*; Sprague-Dawley rats; duodenum	1.0	Activity	Munday and Munday (2004) [ 89]
GST ^e^	Iberin	40 µmol per·kg body wt. for 5 d	*in vivo*; Sprague-Dawley rats; forestomach	1.1	Activity	Munday and Munday (2004) [ 89]
GST ^e^	MIX ^b^	15% broccoli seed/85% RM1 feed for 7 d	*in vivo*; *Nrf2*(+/+) mice; stomach, small intestine, and liver	1.5	Activity	McWalter *et al.* (2004) [78]
GST ^e^	MIX ^b^	Brussels sprouts extract (7 g tissue) for 4 d	*in vivo*; Wistar rats; hepatic cells	1.3	Expression	Sorensen *et al.* (2001) [90]
GST ^e^	MIX ^b^	40 µmol ITC per kg body wt. for 14 d	*in vivo*; Sprague-Dawley rats; bladder	1.4	Activity	Zhang *et al.* (2006) [91]
GST ^e^	MIX ^b^	160 µmol ITC per kg body wt. for 14 d	*in vivo*; Sprague-Dawley rats; bladder	2.1	Activity	Zhang *et al.* (2006) [91]
GST ^e^	MIX ^b^	40 µmol ITC per kg body wt. for 14 d	*in vivo*; Sprague-Dawley rats; duodenum	1.5	Activity	Zhang *et al.* (2006) [91]
GST ^e^	MIX ^b^	160 µmol ITC per kg body wt. for 14 d	*in vivo*; Sprague-Dawley rats; duodenum	2.8	Activity	Zhang *et al.* (2006) [91]
GST ^e^	SF	5 µM	*in vitro*; rat cardiomyocytes	2–2.5 ^d^	Activity	Angeloni *et al.* (2009) [82]
GST ^e^	SF	5 µM	*in vitro*; rat cardiomyocytes	3–5 ^d^	Protein	Angeloni *et al.* (2009) [82]
GST ^e^	SF	1 µM	*in vitro*; rat hepatic Clone 9 cells	2	Activity	Lii *et al.* (2010) [84]
GST ^e^	SF	5 µM	*in vitro*; rat hepatic Clone 9 cells	2.6	Activity	Lii *et al.* (2010) [84]
GST ^e^	SF	3 µmol/g supplemented RM1 feed	*in vivo*; Nrf2(+/+) mice; intestinal cytosol	1.5	Activity	McMahon *et al.* (2001) [87]
GST ^e^	SF	40 µmol per kg body wt. for 5 d	*in vivo*; Sprague-Dawley rats; bladder	2.5	Activity	Munday and Munday (2004) [ 89]
GST ^e^	SF	40 µmol per kg body wt. for 5 d	*in vivo*; Sprague-Dawley rats; duodenum	1.3	Activity	Munday and Munday (2004) [ 89]
GST ^e^	SF	40 µmol per kg body wt. for 5 d	*in vivo*; Sprague-Dawley rats; forestomach	1.2	Activity	Munday and Munday (2004) [ 89]
GST ^e^	SF	313 nM	*in vitro*; human BEAS-2B cells	1.1	Activity	Ritz *et al.* (2007) [92]
GST ^e^	SF	625 nM	*in vitro*; human BEAS-2B cells	1.2	Activity	Ritz *et al.* (2007) [92]
GST ^e^	SF	1.25 µM	*in vitro*; human BEAS-2B cells	1.4	Activity	Ritz *et al.* (2007) [92]
GST ^e^	SF	2.5 µM	*in vitro*; human BEAS-2B cells	1.7	Activity	Ritz *et al.* (2007) [92]
GST ^e^	SF	5 µM	*in vitro*; human BEAS-2B cells	2	Activity	Ritz *et al.* (2007) [92]
GST ^e^	SF	10 µM	*in vitro*; human BEAS-2B cells	2.1	Activity	Ritz *et al.* (2007) [92]
GST ^e^	SF	313 nM	*in vitro*; human NHBE cells	1.2	Activity	Ritz *et al.* (2007) [92]
GST ^e^	SF	625 nM	*in vitro*; human NHBE cells	1.7	Activity	Ritz *et al.* (2007) [92]
GST ^e^	SF	1.25 µM	*in vitro*; human NHBE cells	1.9	Activity	Ritz *et al.* (2007) [92]
GST ^e^	SF	2.5 µM	*in vitro*; human NHBE cells	2.5	Activity	Ritz *et al.* (2007) [92]
GST ^e^	SF	5 µM	*in vitro*; human NHBE cells	2.6	Activity	Ritz *et al.* (2007) [92]
GST ^e^	SF	10 µM	*in vitro*; human NHBE cells	2.8	Activity	Ritz *et al.* (2007) [92]
GST ^e^	SF	9 µmol per day	*in vivo*; ICR mice; small intestine cells	1.3	Activity	Thimmulappa *et al.* (2002) [79]
GST ^e^	SF	9 µmol per day	*in vivo*; ICR mice; small intestine cells	2.5–6 ^f^	Expression	Thimmulappa *et al.* (2002) [79]
GST ^e^	SF	0.1 µM	*in vitro*; mouse cortical neurons	1.7	Activity	Vauzour *et al.* (2010) [83]
GST ^e^	SF	4 µM	*in vitro*; mouse embryonic fibroblasts	1.5	Activity	Zhang *et al.* (2006) [91]
GST ^e^	SF	8 µM	*in vitro*; mouse embryonic fibroblasts	1.5	Activity	Zhang *et al.* (2006) [91]
GST ^e^	SF	4 µM	*in vitro*; rat bladder NBT-II cells	1.8	Activity	Zhang *et al.* (2006) [91]
GST ^e^	SF	8 µM	*in vitro*; rat bladder NBT-II cells	2.1	Activity	Zhang *et al.* (2006) [91]
GSTM	I3C	2 µM	*in vitro*; HepG2 cells	2	Expression	Krajka-Kuźniak *et al.* (2015) [44]
GSTM	I3C	2 µM	*in vitro*; HepG2 cells	1.2	Protein	Krajka-Kuźniak *et al.* (2015) [44]
GSTM	I3C	10 µM	*in vitro*; HepG2 cells	2.2	Expression	Krajka-Kuźniak *et al.* (2015) [44]
GSTM	I3C	10 µM	*in vitro*; HepG2 cells	1.3	Protein	Krajka-Kuźniak *et al.* (2015) [44]
GSTM	PEITC	1 µM	*in vitro*; HepG2 cells	3	Expression	Krajka-Kuźniak *et al.* (2015) [44]
GSTM	PEITC	1 µM	*in vitro*; HepG2 cells	1.3	Protein	Krajka-Kuźniak *et al.* (2015) [44]
GSTM	PEITC	5 µM	*in vitro*; HepG2 cells	4.2	Expression	Krajka-Kuźniak *et al.* (2015) [44]
GSTM	PEITC	5 µM	*in vitro*; HepG2 cells	1.4	Protein	Krajka-Kuźniak *et al.* (2015) [44]
GSTM	SF	0.2 µM	*in vitro*; rat hepatic Clone 9 cells	1.9	Protein	Lii *et al.* (2010) [84]
GSTM	SF	1 µM	*in vitro*; rat hepatic Clone 9 cells	3.5	Protein	Lii *et al.* (2010) [84]
GSTM	SF	5 µM	*in vitro*; rat hepatic Clone 9 cells	5.0	Protein	Lii *et al.* (2010) [84]
GSTM1	I3C	0.5% (*w*/*w*) supplemented RM1 feed	*in vivo*; Nrf2(+/+) mice; intestinal cytosol	1.9	Protein	McMahon *et al.* (2001) [87]
GSTM1	PEITC	40 mg/kg; 3 h after treatment	*in vivo*; Nrf2(+/+) mice; liver	2.5 (2.3) ^c^	Expression	Hu *et al.* (2006) [88]
GSTM1	PEITC	40 mg/kg; 12 h after treatment	*in vivo*; Nrf2(+/+) mice; liver	1.9 (2.2) ^c^	Expression	Hu *et al.* (2006) [88]
GSTM1	SF	90 mg/kg; 12 h after treatment	*in vivo*; Nrf2(+/+) mice; liver	4.4	Expression	Hu *et al.* (2006) [81]
GSTM1	SF	3 µmol/g supplemented RM1 feed	*in vivo*; Nrf2(+/+) mice; intestinal cytosol	1.4	Protein	McMahon *et al.* (2001) [87]
GSTM1	SF	13 µmol/d for 3 d	Human clinical; nasal lavage cells	0.9	Expression	Riedl *et al.* (2009) [93]
GSTM1	SF	51 µmol/d for 3 d	Human clinical; nasal lavage cells	1.1	Expression	Riedl *et al.* (2009) [93]
GSTM1	SF	64 µmol/d for 3 d	Human clinical; nasal lavage cells	1.3	Expression	Riedl *et al.* (2009) [93]
GSTM1	SF	76 µmol/d for 3 d	Human clinical; nasal lavage cells	1.7	Expression	Riedl *et al.* (2009) [93]
GSTM1	SF	89 µmol/d for 3 d	Human clinical; nasal lavage cells	1.9	Expression	Riedl *et al.* (2009) [93]
GSTM1	SF	102 µmol/d for 3 d	Human clinical; nasal lavage cells	2.2	Expression	Riedl *et al.* (2009) [93]
GSTM1	SF	5 µM	*in vitro*; human BEAS-2B cells	1	Expression	Ritz *et al.* (2007) [92]
GSTM1	SF	5 µM	*in vitro*; human NHBE cells	2	Expression	Ritz *et al.* (2007) [92]
GSTM3	PEITC	40 mg/kg; 12 h after treatment	*in vivo*; Nrf2(+/+) mice; liver	2	Expression	Hu *et al.* (2006) [88]
GSTM5	I3C	0.5% (*w*/*w*) supplemented RM1 feed	*in vivo*; Nrf2(+/+) mice; intestinal cytosol	5.1	Protein	McMahon *et al.* (2001) [87]
GSTM5	SF	90 mg/kg; 12 h after treatment	*in vivo*; Nrf2(+/+) mice; liver	2	Expression	Hu *et al.* (2006) [81]
GSTM5	SF	3 µmol/g supplemented RM1 feed	*in vivo*; Nrf2(+/+) mice; intestinal cytosol	0.9	Protein	McMahon *et al.* (2001) [87]
GSTP	DIM	5 µM	*in vitro*; MCF10A breast cells	0.5	Expression	Szaefer *et al.* (2015) [94]
GSTP	DIM	10 µM	*in vitro*; MCF10A breast cells	0.4	Expression	Szaefer *et al.* (2015) [94]
GSTP	DIM	5 µM	*in vitro*; MCF10A breast cells	1	Protein	Szaefer *et al.* (2015) [94]
GSTP	DIM	10 µM	*in vitro*; MCF10A breast cells	0.8	Protein	Szaefer *et al.* (2015) [94]
GSTP	DIM	5 µM	*in vitro*; MCF7 breast cells	1.2	Expression	Szaefer *et al.* (2015) [94]
GSTP	DIM	10 µM	*in vitro*; MCF7 breast cells	1.8	Expression	Szaefer *et al.* (2015) [94]
GSTP	DIM	5 µM	*in vitro*; MCF7 breast cells	1.1	Protein	Szaefer *et al.* (2015) [94]
GSTP	DIM	10 µM	*in vitro*; MCF7 breast cells	1	Protein	Szaefer *et al.* (2015) [94]
GSTP	DIM	5 µM	*in vitro*; MDA-MB-231 breast cells	1.2	Expression	Szaefer *et al.* (2015) [94]
GSTP	DIM	10 µM	*in vitro*; MDA-MB-231 breast cells	1.8	Expression	Szaefer *et al.* (2015) [94]
GSTP	DIM	5 µM	*in vitro*; MDA-MB-231 breast cells	1	Protein	Szaefer *et al.* (2015) [94]
GSTP	DIM	10 µM	*in vitro*; MDA-MB-231 breast cells	1.1	Protein	Szaefer *et al.* (2015) [94]
GSTP	I3C	2 µM	*in vitro*; HepG2 cells	2.8	Expression	Krajka-Kuźniak *et al.* (2015) [44]
GSTP	I3C	10 µM	*in vitro*; HepG2 cells	3.3	Expression	Krajka-Kuźniak *et al.* (2015) [44]
GSTP	I3C	2 µM	*in vitro*; HepG2 cells	1.2	Protein	Krajka-Kuźniak *et al.* (2015) [44]
GSTP	I3C	10 µM	*in vitro*; HepG2 cells	1.3	Protein	Krajka-Kuźniak *et al.* (2015) [44]
GSTP	I3C	10 µM	*in vitro*; MCF10A breast cells	0.5	Expression	Szaefer *et al.* (2015) [94]
GSTP	I3C	50 µM	*in vitro*; MCF10A breast cells	0.4	Expression	Szaefer *et al.* (2015) [94]
GSTP	I3C	10 µM	*in vitro*; MCF10A breast cells	1	Protein	Szaefer *et al.* (2015) [94]
GSTP	I3C	50 µM	*in vitro*; MCF10A breast cells	0.9	Protein	Szaefer *et al.* (2015) [94]
GSTP	I3C	30 µM	*in vitro*; MCF7 breast cells	1.5	Expression	Szaefer *et al.* (2015) [94]
GSTP	I3C	50 µM	*in vitro*; MCF7 breast cells	1.8	Expression	Szaefer *et al.* (2015) [94]
GSTP	I3C	30 µM	*in vitro*; MCF7 breast cells	1.2	Protein	Szaefer *et al.* (2015) [94]
GSTP	I3C	50 µM	*in vitro*; MCF7 breast cells	1.3	Protein	Szaefer *et al.* (2015) [94]
GSTP	I3C	10 µM	*in vitro*; MDA-MB-231 breast cells	1	Expression	Szaefer *et al.* (2015) [94]
GSTP	I3C	50 µM	*in vitro*; MDA-MB-231 breast cells	1.5	Expression	Szaefer *et al.* (2015) [94]
GSTP	I3C	10 µM	*in vitro*; MDA-MB-231 breast cells	1	Protein	Szaefer *et al.* (2015) [94]
GSTP	I3C	50 µM	*in vitro*; MDA-MB-231 breast cells	1	Protein	Szaefer *et al.* (2015) [94]
GSTP	PEITC	1 µM	*in vitro*; HepG2 cells	2.7	Expression	Krajka-Kuźniak *et al.* (2015) [44]
GSTP	PEITC	5 µM	*in vitro*; HepG2 cells	5	Expression	Krajka-Kuźniak *et al.* (2015) [44]
GSTP	PEITC	1 µM	*in vitro*; HepG2 cells	1.2	Protein	Krajka-Kuźniak *et al.* (2015) [44]
GSTP	PEITC	5 µM	*in vitro*; HepG2 cells	1.4	Protein	Krajka-Kuźniak *et al.* (2015) [44]
GSTP	SF	1 µM	*in vitro*; rat hepatic Clone 9 cells	1	Expression	Lii *et al.* (2010) [84]
GSTP	SF	5 µM	*in vitro*; rat hepatic Clone 9 cells	5	Expression	Lii *et al.* (2010) [84]
GSTP	SF	0.2 µM	*in vitro*; rat hepatic Clone 9 cells	5	Protein	Lii *et al.* (2010) [84]
GSTP	SF	1 µM	*in vitro*; rat hepatic Clone 9 cells	7	Protein	Lii *et al.* (2010) [84]
GSTP	SF	5 µM	*in vitro*; rat hepatic Clone 9 cells	8.1	Protein	Lii *et al.* (2010) [84]
GSTP1	MIX ^b^	Broccoli sprout extract; 0.5 mg/L	*in vitro*; human A549 cells	1.5 (24 h) ^g^	Expression	Tan *et al.* (2010) [95]
GSTP1	MIX ^b^	Broccoli sprout extract; 1 mg/L	*in vitro*; human A549 cells	1.6 (24 h) ^g^	Expression	Tan *et al.* (2010) [95]
GSTP1	MIX ^b^	Broccoli sprout extract; 2 mg/L	*in vitro*; human A549 cells	2.5 (24 h) ^g^	Expression	Tan *et al.* (2010) [95]
GSTP1	MIX ^b^	Broccoli sprout extract; 2 mg/L	*in vitro*; human immortalized HBE cells	3.2 (24 h) ^g^	Expression	Tan *et al.* (2010) [95]
GSTP1	MIX ^b^	Broccoli sprout extract; 2 mg/L	*in vitro*; human NHBE cells	2 (48 h) ^g^	Expression	Tan *et al.* (2010) [95]
GSTP1	SF	13 µmol/d for 3 d	Human clinical; nasal lavage cells	1	Expression	Riedl *et al.* (2009) [93]
GSTP1	SF	51 µmol/d for 3 d	Human clinical; nasal lavage cells	1.1	Expression	Riedl *et al.* (2009) [93]
GSTP1	SF	64 µmol/d for 3 d	Human clinical; nasal lavage cells	1.4	Expression	Riedl *et al.* (2009) [93]
GSTP1	SF	76 µmol/d for 3 d	Human clinical; nasal lavage cells	1.8	Expression	Riedl *et al.* (2009) [93]
GSTP1	SF	89 µmol/d for 3 d	Human clinical; nasal lavage cells	1.9	Expression	Riedl *et al.* (2009) [93]
GSTP1	SF	102 µmol/d for 3 d	Human clinical; nasal lavage cells	2	Expression	Riedl *et al.* (2009) [93]
GSTP1/2	I3C	0.5% (*w*/*w*) supplemented RM1 feed	*in vivo*; Nrf2(+/+) mice; intestinal cytosol	1	Protein	McMahon *et al.* (2001) [87]
GSTP1/2	SF	3 µmol/g supplemented RM1 feed	*in vivo*; Nrf2(+/+) mice; intestinal cytosol	0.9	Protein	McMahon *et al.* (2001) [87]
GSTT	I3C	2 µM	*in vitro*; HepG2 cells	1.3	Expression	Krajka-Kuźniak *et al.* (2015) [44]
GSTT	I3C	10 µM	*in vitro*; HepG2 cells	1.2	Expression	Krajka-Kuźniak *et al.* (2015) [44]
GSTT	I3C	2 µM	*in vitro*; HepG2 cells	1.2	Protein	Krajka-Kuźniak *et al.* (2015) [44]
GSTT	I3C	10 µM	*in vitro*; HepG2 cells	1.2	Protein	Krajka-Kuźniak *et al.* (2015) [44]
GSTT	PEITC	1 µM	*in vitro*; HepG2 cells	1.5	Expression	Krajka-Kuźniak *et al.* (2015) [44]
GSTT	PEITC	5 µM	*in vitro*; HepG2 cells	2.5	Expression	Krajka-Kuźniak *et al.* (2015) [44]
GSTT	PEITC	1 µM	*in vitro*; HepG2 cells	1.3	Protein	Krajka-Kuźniak *et al.* (2015) [44]
GSTT	PEITC	5 µM	*in vitro*; HepG2 cells	1.3	Protein	Krajka-Kuźniak *et al.* (2015) [44]
GSTT3	PEITC	40 mg/kg; 3 h after treatment	*in vivo*; Nrf2(+/+) mice; liver	2.8	Expression	Hu *et al.* (2006) [88]
HO-1	AITC	5 µM	*in vitro*; NIH3T3 cells	10	Expression	Ernst *et al.* (2011) [74]
HO-1	AITC	10 µM	*in vitro*; NIH3T3 cells	20	Expression	Ernst *et al.* (2011) [74]
HO-1	AITC	25 µM	*in vitro*; NIH3T3 cells	45	Expression	Ernst *et al.* (2011) [74]
HO-1	BITC	5 µM	*in vitro*; NIH3T3 cells	5	Expression	Ernst *et al.* (2011) [74]
HO-1	BITC	10 µM	*in vitro*; NIH3T3 cells	11	Expression	Ernst *et al.* (2011) [74]
HO-1	BITC	25 µM	*in vitro*; NIH3T3 cells	24	Expression	Ernst *et al.* (2011) [74]
HO-1	DIM	5 µM	*in vitro*; NIH3T3 cells	4.3	Expression	Ernst *et al.* (2011) [75]
HO-1	DIM	10 µM	*in vitro*; NIH3T3 cells	7	Expression	Ernst *et al.* (2011) [75]
HO-1	DIM	25 µM	*in vitro*; NIH3T3 cells	13	Expression	Ernst *et al.* (2011) [75]
HO-1	Erucin	25 µM precursor + myrosinase; 6 h treatment	*in vitro*; HT-29 cells	290	Expression	Wagner *et al.* (2015) [96]
HO-1	Erucin	20 mg/kg precursor + myrosinase for 7 d	*in vivo*; C57BL/6 mice; brain	1.1	Expression	Wagner *et al.* (2015) [96]
HO-1	Erucin	20 mg/kg precursor + myrosinase for 7 d	*in vivo*; C57BL/6 mice; liver	3	Expression	Wagner *et al.* (2015) [96]
HO-1	Erucin	20 mg/kg precursor + myrosinase for 7 d	*in vivo*; C57BL/6 mice; mucosae	4.8	Expression	Wagner *et al.* (2015) [96]
HO-1	I3C	25 µM	*in vitro*; TRAMP C1 cells	1.5	Expression	Wu *et al.* (2012) [77]
HO-1	I3C	50 µM	*in vitro*; TRAMP C1 cells	1.8	Expression	Wu *et al.* (2012) [77]
HO-1	I3C	75 µM	*in vitro*; TRAMP C1 cells	2.8	Expression	Wu *et al.* (2012) [77]
HO-1	PEITC	5 µM	*in vitro*; NIH3T3 cells	33	Expression	Ernst *et al.* (2011) [74]
HO-1	SF	5 µM	*in vitro*; NIH3T3 cells	18.6	Expression	Ernst *et al.* (2011) [75]
HO-1	SF	10 µM	*in vitro*; NIH3T3 cells	27	Expression	Ernst *et al.* (2011) [75]
HO-1	SF	5 µM	*in vitro*; NIH3T3 cells	18	Expression	Ernst *et al.* (2011) [74]
HO-1	SF	10 µM	*in vitro*; NIH3T3 cells	27	Expression	Ernst *et al.* (2011) [74]
HO-1	SF	90 mg/kg; 3 h after treatment	*in vivo*; Nrf2(+/+) mice; liver	10.3 (12.2) ^c^	Expression	Hu *et al.* (2006) [81]
HO-1	SF	13 µmol/d for 3 d	Human clinical; nasal lavage cells	1	Expression	Riedl *et al.* (2009) [93]
HO-1	SF	64 µmol/d for 3 d	Human clinical; nasal lavage cells	1.4	Expression	Riedl *et al.* (2009) [93]
HO-1	SF	76 µmol/d for 3 d	Human clinical; nasal lavage cells	2.1	Expression	Riedl *et al.* (2009) [93]
HO-1	SF	89 µmol/d for 3 d	Human clinical; nasal lavage cells	2.1	Expression	Riedl *et al.* (2009) [93]
HO-1	SF	102 µmol/d for 3 d	Human clinical; nasal lavage cells	2.2	Expression	Riedl *et al.* (2009) [93]
HO-1	SF	50 µM	*in vitro*; human Caco-2 cells	3.8	Expression	Traka *et al.* (2005) [97]
NQO1	AITC	5 µM	*in vitro*; NIH3T3 cells	2.2	Expression	Ernst *et al.* (2011) [74]
NQO1	AITC	10 µM	*in vitro*; NIH3T3 cells	2.1	Expression	Ernst *et al.* (2011) [74]
NQO1	AITC	25 µM	*in vitro*; NIH3T3 cells	1.9	Expression	Ernst *et al.* (2011) [74]
NQO1	AITC	40 µmol per kg body wt. for 5 d	*in vivo*; Sprague-Dawley rats; bladder	1.9	Activity	Munday and Munday (2004) [ 89]
NQO1	AITC	40 µmol per kg body wt. for 5 d	*in vivo*; Sprague-Dawley rats; duodenum	1.5	Activity	Munday and Munday (2004) [ 89]
NQO1	AITC	40 µmol per kg body wt. for 5 d	*in vivo*; Sprague-Dawley rats; forestomach	1.6	Activity	Munday and Munday (2004) [ 89]
NQO1	BITC	50 µM	*in vitro*; human LS-174 cells	1.5	Activity	Bonnesen *et al.* (2001) [98]
NQO1	BITC	50 µM	*in vitro*; human LS-174 cells	15–20 ^h^	Protein	Bonnesen *et al.* (2001) [98]
NQO1	BITC	5 µM	*in vitro*; NIH3T3 cells	2	Expression	Ernst *et al.* (2011) [74]
NQO1	BITC	10 µM	*in vitro*; NIH3T3 cells	1.7	Expression	Ernst *et al.* (2011) [74]
NQO1	BITC	25 µM	*in vitro*; NIH3T3 cells	1.6	Expression	Ernst *et al.* (2011) [74]
NQO1	BITC	2 µM	*in vitro*; human NHBE cells	7.5 (24 h) ^g^	Protein	Tan *et al.* (2010) [95]
NQO1	DIM	300 µM	*in vitro*; human LS-174 cells	1.1	Activity	Bonnesen *et al.* (2001) [98]
NQO1	DIM	300 µM	*in vitro*; human LS-174 cells	2	Protein	Bonnesen *et al.* (2001) [98]
NQO1	DIM	5 µM	*in vitro*; NIH3T3 cells	1.7	Expression	Ernst *et al.* (2011) [75]
NQO1	DIM	10 µM	*in vitro*; NIH3T3 cells	1.7	Expression	Ernst *et al.* (2011) [75]
NQO1	DIM	25 µM	*in vitro*; NIH3T3 cells	2	Expression	Ernst *et al.* (2011) [75]
NQO1	DIM	5 µM	*in vitro*; MCF10A breast cells	1.3	Expression	Szaefer *et al.* (2015) [94]
NQO1	DIM	10 µM	*in vitro*; MCF10A breast cells	1.5	Expression	Szaefer *et al.* (2015) [94]
NQO1	DIM	5 µM	*in vitro*; MCF10A breast cells	1	Protein	Szaefer *et al.* (2015) [94]
NQO1	DIM	10 µM	*in vitro*; MCF10A breast cells	1	Protein	Szaefer *et al.* (2015) [94]
NQO1	DIM	5 µM	*in vitro*; MCF7 breast cells	2.3	Expression	Szaefer *et al.* (2015) [94]
NQO1	DIM	10 µM	*in vitro*; MCF7 breast cells	3.8	Expression	Szaefer *et al.* (2015) [94]
NQO1	DIM	5 µM	*in vitro*; MCF7 breast cells	1	Protein	Szaefer *et al.* (2015) [94]
NQO1	DIM	10 µM	*in vitro*; MCF7 breast cells	1.1	Protein	Szaefer *et al.* (2015) [94]
NQO1	DIM	5 µM	*in vitro*; MDA-MB-231 breast cells	4.1	Expression	Szaefer *et al.* (2015) [94]
NQO1	DIM	10 µM	*in vitro*; MDA-MB-231 breast cells	5.1	Expression	Szaefer *et al.* (2015) [94]
NQO1	DIM	5 µM	*in vitro*; MDA-MB-231 breast cells	1	Protein	Szaefer *et al.* (2015) [94]
NQO1	DIM	10 µM	*in vitro*; MDA-MB-231 breast cells	1.1	Protein	Szaefer *et al.* (2015) [94]
NQO1	Erucin	40 µmol per kg body wt. for 5 d	*in vivo*; Sprague-Dawley rats; bladder	1.7	Activity	Munday and Munday (2004) [ 89]
NQO1	Erucin	40 µmol per kg body wt. for 5 d	*in vivo*; Sprague-Dawley rats; duodenum	1.5	Activity	Munday and Munday (2004) [ 89]
NQO1	Erucin	40 µmol per kg body wt. for 5 d	*in vivo*; Sprague-Dawley rats; forestomach	1.3	Activity	Munday and Munday (2004) [ 89]
NQO1	I3C	1 mM	*in vitro*; human LS-174 cells	1.1	Activity	Bonnesen *et al.* (2001) [98]
NQO1	I3C	1 mM	*in vitro*; human LS-174 cells	2	Protein	Bonnesen *et al.* (2001) [98]
NQO1	I3C	0.5% (*w*/*w*) supplemented RM1 feed	*in vivo*; Nrf2(+/+) mice; intestinal cytosol	1.4	Activity	McMahon *et al.* (2001) [87]
NQO1	I3C	0.5% (*w*/*w*) supplemented RM1 feed	*in vivo*; Nrf2(+/+) mice; intestinal cytosol	2.4	Protein	McMahon *et al.* (2001) [87]
NQO1	I3C	10 µM	*in vitro*; MCF10A breast cells	1.2	Expression	Szaefer *et al.* (2015) [94]
NQO1	I3C	50 µM	*in vitro*; MCF10A breast cells	1.8	Expression	Szaefer *et al.* (2015) [94]
NQO1	I3C	10 µM	*in vitro*; MCF10A breast cells	1.1	Protein	Szaefer *et al.* (2015) [94]
NQO1	I3C	50 µM	*in vitro*; MCF10A breast cells	1.1	Protein	Szaefer *et al.* (2015) [94]
NQO1	I3C	30 µM	*in vitro*; MCF7 breast cells	2	Expression	Szaefer *et al.* (2015) [94]
NQO1	I3C	50 µM	*in vitro*; MCF7 breast cells	2.7	Expression	Szaefer *et al.* (2015) [94]
NQO1	I3C	30 µM	*in vitro*; MCF7 breast cells	1.3	Protein	Szaefer *et al.* (2015) [94]
NQO1	I3C	50 µM	*in vitro*; MCF7 breast cells	1.7	Protein	Szaefer *et al.* (2015) [94]
NQO1	I3C	10 µM	*in vitro*; MDA-MB-231 breast cells	2	Expression	Szaefer *et al.* (2015) [94]
NQO1	I3C	50 µM	*in vitro*; MDA-MB-231 breast cells	3	Expression	Szaefer *et al.* (2015) [94]
NQO1	I3C	10 µM	*in vitro*; MDA-MB-231 breast cells	1	Protein	Szaefer *et al.* (2015) [94]
NQO1	I3C	50 µM	*in vitro*; MDA-MB-231 breast cells	1.2	Protein	Szaefer *et al.* (2015) [94]
NQO1	I3C	25 µM	*in vitro*; TRAMP C1 cells	1.7	Expression	Wu *et al.* (2012) [77]
NQO1	I3C	50 µM	*in vitro*; TRAMP C1 cells	2.7	Expression	Wu *et al.* (2012) [77]
NQO1	I3C	75 µM	*in vitro*; TRAMP C1 cells	3.8	Expression	Wu *et al.* (2012) [77]
NQO1	Iberin	40 µmol per kg body wt. for 5 d	*in vivo*; Sprague-Dawley rats; bladder	2.2	Activity	Munday and Munday (2004) [ 89]
NQO1	Iberin	40 µmol per kg body wt. for 5 d	*in vivo*; Sprague-Dawley rats; duodenum	1.8	Activity	Munday and Munday (2004) [ 89]
NQO1	Iberin	40 µmol per kg body wt. for 5 d	*in vivo*; Sprague-Dawley rats; forestomach	1.3	Activity	Munday and Munday (2004) [ 89]
NQO1	MIX ^b^	15% broccoli seed/85% RM1 feed for 7 d	*in vitro*; mouse Hepa 1c1c7 cells	3	Activity	McWalter *et al.* (2004) [78]
NQO1	MIX ^b^	15% broccoli seed/85% RM1 feed for 7 d	*in vitro*; rat RL-34 cells	5	Activity	McWalter *et al.* (2004) [78]
NQO1	MIX ^b^	15% broccoli seed/85% RM1 feed for 7 d	*in vivo*; Nrf2(+/+) mice; stomach, small intestine, and liver	1.5	Activity	McWalter *et al.* (2004) [78]
NQO1	MIX ^b^	15% broccoli seed/85% RM1 feed for 7 d	*in vivo*; Nrf2(+/+) mice; stomach, small intestine, and liver	2	Protein	McWalter *et al.* (2004) [78]
NQO1	MIX ^b^	Brussels sprouts extract (7 g tissue) for 4d	*in vivo*; Wistar rats; hepatic cells	2.6	Activity	Sorensen *et al.* (2001) [90]
NQO1	MIX ^b^	Broccoli sprout extract; 2 mg/L	*in vitro*; human A549 cells	1.9 (24 h) ^g^	Expression	Tan *et al.* (2010) [95]
NQO1	MIX ^b^	Broccoli sprout extract; 2 mg/L	*in vitro*; human immortalized HBE cells	4 (6d) ^g^	Expression	Tan *et al.* (2010) [95]
NQO1	MIX ^b^	Broccoli sprout extract; 1 mg/L	*in vitro*; human NHBE cells	2.1 (24 h) ^g^	Expression	Tan *et al.* (2010) [95]
NQO1	MIX ^b^	Broccoli sprout extract; 2 mg/L	*in vitro*; human NHBE cells	4.5 (24 h) ^g^	Expression	Tan *et al.* (2010) [95]
NQO1	MIX ^b^	Broccoli sprout extract; 2 mg/L	*in vitro*; human NHBE cells	5 (24 h) ^g^	Protein	Tan *et al.* (2010) [95]
NQO1	MIX ^b^	40 µmol ITC per kg body wt for 14 d	*in vivo*; Sprague-Dawley rats; bladder	2.4	Activity	Zhang *et al.* (2006) [91]
NQO1	MIX ^b^	160 µmol ITC per kg body wt for 14 d	*in vivo*; Sprague-Dawley rats; bladder	4.4	Activity	Zhang *et al.* (2006) [91]
NQO1	MIX ^b^	40 µmol ITC per kg body wt for 14 d	*in vivo*; Sprague-Dawley rats; duodenum	2.4	Activity	Zhang *et al.* (2006) [91]
NQO1	MIX ^b^	160 µmol ITC per kg body wt for 14 d	*in vivo*; Sprague-Dawley rats; duodenum	4.6	Activity	Zhang *et al.* (2006) [91]
NQO1	PEITC	50 µM	*in vitro*; human LS-174 cells	1.4	Activity	Bonnesen *et al.* (2001) [98]
NQO1	PEITC	50 µM	*in vitro*; human LS-174 cells	15–20 ^h^	Protein	Bonnesen *et al.* (2001) [98]
NQO1	PEITC	5 µM	*in vitro*; NIH3T3 cells	1.7	Expression	Ernst *et al.* (2011) [74]
NQO1	PEITC	2 µM	*in vitro*; human immortalized HBE cells	6 (48 h) ^g^	Protein	Tan *et al.* (2010) [95]
NQO1	PEITC	2 µM	*in vitro*; human NHBE cells	10 (6d) ^g^	Protein	Tan *et al.* (2010) [95]
NQO1	SF	5 µM	*in vitro*; rat cardiomyocytes	3–5 ^d^	Activity	Angeloni *et al.* (2009) [82]
NQO1	SF	5 µM	*in vitro*; rat cardiomyocytes	1.5–2.2 ^d^	Expression	Angeloni *et al.* (2009) [82]
NQO1	SF	5 µM	*in vitro*; rat cardiomyocytes	2–3 ^d^	Protein	Angeloni *et al.* (2009) [82]
NQO1	SF	50 µM	*in vitro*; human LS-174 cells	2	Activity	Bonnesen *et al.* (2001) [98]
NQO1	SF	50 µM	*in vitro*; human LS-174 cells	15–20 ^h^	Protein	Bonnesen *et al.* (2001) [98]
NQO1	SF	0.1 µM	*in vitro*; human prostatic cancer cells	1 ^g^	Activity	Brooks *et al.* (2001) [85]
NQO1	SF	0.5 µM	*in vitro*; human prostatic cancer cells	1.1 ^g^	Activity	Brooks *et al.* (2001) [85]
NQO1	SF	1 µM	*in vitro*; human prostatic cancer cells	1.3 ^i^	Activity	Brooks *et al.* (2001) [85]
NQO1	SF	5 µM	*in vitro*; human prostatic cancer cells	1.8 ^i^	Activity	Brooks *et al.* (2001) [85]
NQO1	SF	8 µM	*in vitro*; human prostatic cancer cells	1.9 ^i^	Activity	Brooks *et al.* (2001) [85]
NQO1	SF	10 µM	*in vitro*; human prostatic cancer cells	1.9 ^i^	Activity	Brooks *et al.* (2001) [85]
NQO1	SF	15 µM	*in vitro*; human prostatic cancer cells	1.8 ^i^	Activity	Brooks *et al.* (2001) [85]
NQO1	SF	10 µM	*in vitro*; human prostatic cancer cells (LNCaP)	2.6	Expression	Brooks *et al.* (2001) [85]
NQO1	SF	10 µM	*in vitro*; human prostatic cancer cells (MDA Pca 2A)	2.2	Expression	Brooks *et al.* (2001) [85]
NQO1	SF	10 µM	*in vitro*; human prostatic cancer cells (MDA Pca 2B)	1.9	Expression	Brooks *et al.* (2001) [85]
NQO1	SF	10 µM	*in vitro*; human prostatic cancer cells (PC3)	1.8	Expression	Brooks *et al.* (2001) [85]
NQO1	SF	10 µM	*in vitro*; human prostatic cancer cells (TSU-Pr1)	1.6	Expression	Brooks *et al.* (2001) [85]
NQO1	SF	0.1 µM	*in vitro*; normal human prostatic cells	1.4	Activity	Brooks *et al.* (2001) [85]
NQO1	SF	0.5 µM	*in vitro*; normal human prostatic cells	1.6	Activity	Brooks *et al.* (2001) [85]
NQO1	SF	1 µM	*in vitro*; normal human prostatic cells	2.1	Activity	Brooks *et al.* (2001) [85]
NQO1	SF	3 µM	*in vitro*; normal human prostatic cells	2.5	Activity	Brooks *et al.* (2001) [85]
NQO1	SF	5 µM	*in vitro*; normal human prostatic cells	2	Activity	Brooks *et al.* (2001) [85]
NQO1	SF	8 µM	*in vitro*; normal human prostatic cells	1.8	Activity	Brooks *et al.* (2001) [85]
NQO1	SF	10 µM	*in vitro*; normal human prostatic cells	1.9	Activity	Brooks *et al.* (2001) [85]
NQO1	SF	15 µM	*in vitro*; normal human prostatic cells	1.8	Activity	Brooks *et al.* (2001) [85]
NQO1	SF	40 nmol	Human clinical; skin	1	Activity	Dinkova-Kostova *et al.* (2007) [99]
NQO1	SF	170 nmol	Human clinical; skin	1.5	Activity	Dinkova-Kostova *et al.* (2007) [99]
NQO1	SF	340 nmol	Human clinical; skin	1.6	Activity	Dinkova-Kostova *et al.* (2007) [99]
NQO1	SF	50 nmol/d for 3 d	Human clinical; skin	2.8	Activity	Dinkova-Kostova *et al.* (2007) [99]
NQO1	SF	100 nmol/d for 3 d	Human clinical; skin	3	Activity	Dinkova-Kostova *et al.* (2007) [99]
NQO1	SF	150 nmol/d for 3 d	Human clinical; skin	4.5	Activity	Dinkova-Kostova *et al.* (2007) [99]
NQO1	SF	200 nmol/d for 3 d	Human clinical; skin	2.7	Activity	Dinkova-Kostova *et al.* (2007) [99]
NQO1	SF	100 nmol/cm^2^; 1 dose	*in vivo*; SKH-1 hairless mice; skin	1.6	Activity	Dinkova-Kostova *et al.* (2007) [99]
NQO1	SF	100 nmol/cm^2^; 3 doses (1/d for 3 d)	*in vivo*; SKH-1 hairless mice; skin	2.7	Activity	Dinkova-Kostova *et al.* (2007) [99]
NQO1	SF	5 µM	*in vitro*; NIH3T3 cells	2.3	Expression	Ernst *et al.* (2011) [74]
NQO1	SF	10 µM	*in vitro*; NIH3T3 cells	2.2	Expression	Ernst *et al.* (2011) [74]
NQO1	SF	5 µM	*in vitro*; NIH3T3 cells	2.3	Expression	Ernst *et al.* (2011) [75]
NQO1	SF	10 µM	*in vitro*; NIH3T3 cells	2.2	Expression	Ernst *et al.* (2011) [75]
NQO1	SF	156 nM	*in vitro*; human ARPE-19 cells	1.1	Activity	Gao *et al.* (2001) [100]
NQO1	SF	313 nM	*in vitro*; human ARPE-19 cells	1.4	Activity	Gao *et al.* (2001) [100]
NQO1	SF	625 nM	*in vitro*; human ARPE-19 cells	1.6	Activity	Gao *et al.* (2001) [100]
NQO1	SF	1.25 µM	*in vitro*; human ARPE-19 cells	1.8	Activity	Gao *et al.* (2001) [100]
NQO1	SF	2.5 µM	*in vitro*; human ARPE-19 cells	2.0	Activity	Gao *et al.* (2001) [100]
NQO1	SF	5 µM	*in vitro*; human ARPE-19 cells	2.2	Activity	Gao *et al.* (2001) [100]
NQO1	SF	156 nM	*in vitro*; human ARPE-19 cells	1.4	Activity	Gao *et al.* (2004) [101]
NQO1	SF	313 nM	*in vitro*; human ARPE-19 cells	1.7	Activity	Gao *et al.* (2004) [101]
NQO1	SF	625 nM	*in vitro*; human ARPE-19 cells	2.1	Activity	Gao *et al.* (2004) [101]
NQO1	SF	1.25 µM	*in vitro*; human ARPE-19 cells	2.6	Activity	Gao *et al.* (2004) [101]
NQO1	SF	2.5 µM	*in vitro*; human ARPE-19 cells	3.3	Activity	Gao *et al.* (2004) [101]
NQO1	SF	0.2 µM	*in vitro*; rat hepatic Clone 9 cells	5.1	Protein	Lii *et al.* (2010) [84]
NQO1	SF	1 µM	*in vitro*; rat hepatic Clone 9 cells	6.6	Protein	Lii *et al.* (2010) [84]
NQO1	SF	5 µM	*in vitro*; rat hepatic Clone 9 cells	3.9	Activity	Lii *et al.* (2010) [84]
NQO1	SF	5 µM	*in vitro*; rat hepatic Clone 9 cells	7.8	Protein	Lii *et al.* (2010) [84]
NQO1	SF	3 µmol/g supplemented RM1 feed	*in vivo*; Nrf2(+/+) mice; intestinal cytosol	1.4	Activity	McMahon *et al.* (2001) [87]
NQO1	SF	3 µmol/g supplemented RM1 feed	*in vivo*; Nrf2(+/+) mice; intestinal cytosol	1.2	Protein	McMahon *et al.* (2001) [87]
NQO1	SF	5 µM	*in vitro*; mouse Hepa 1c1c7 cells	4.5	Activity	McWalter *et al.* (2004) [78]
NQO1	SF	5 µM	*in vitro*; rat RL-34 cells	5.2	Activity	McWalter *et al.* (2004) [78]
NQO1	SF	40 µmol per kg body wt. for 5 d	*in vivo*; Sprague-Dawley rats; bladder	1.9	Activity	Munday and Munday (2004) [ 89]
NQO1	SF	40 µmol per kg body wt. for 5 d	*in vivo*; Sprague-Dawley rats; duodenum	2.2	Activity	Munday and Munday (2004) [ 89]
NQO1	SF	40 µmol per kg body wt. for 5 d	*in vivo*; Sprague-Dawley rats; forestomach	1.2	Activity	Munday and Munday (2004) [ 89]
NQO1	SF	13 µmol/d for 3 d	Human clinical; nasal lavage cells	1	Expression	Riedl *et al.* (2009) [93]
NQO1	SF	51 µmol/d for 3 d	Human clinical; nasal lavage cells	1.1	Expression	Riedl *et al.* (2009) [93]
NQO1	SF	64 µmol/d for 3 d	Human clinical; nasal lavage cells	1.5	Expression	Riedl *et al.* (2009) [93]
NQO1	SF	76 µmol/d for 3 d	Human clinical; nasal lavage cells	2.4	Expression	Riedl *et al.* (2009) [93]
NQO1	SF	89 µmol/d for 3 d	Human clinical; nasal lavage cells	2.6	Expression	Riedl *et al.* (2009) [93]
NQO1	SF	102 µmol/d for 3 d	Human clinical; nasal lavage cells	3	Expression	Riedl *et al.* (2009) [93]
NQO1	SF	5 µM	*in vitro*; human BEAS-2B cells	15	Expression	Ritz *et al.* (2007) [92]
NQO1	SF	5 µM	*in vitro*; human NHBE cells	3	Expression	Ritz *et al.* (2007) [92]
NQO1	SF	1 µM	*in vitro*; human immortalized HBE cells	2 (24 h) ^g^	Expression	Tan *et al.* (2010) [95]
NQO1	SF	2 µM	*in vitro*; human immortalized HBE cells	8 (48 h) ^g^	Protein	Tan *et al.* (2010) [95]
NQO1	SF	0.5 µM	*in vitro*; human NHBE cells	3.5 (24 h) ^g^	Expression	Tan *et al.* (2010) [95]
NQO1	SF	1 µM	*in vitro*; human NHBE cells	3.8 (24 h) ^g^	Expression	Tan *et al.* (2010) [95]
NQO1	SF	2 µM	*in vitro*; human NHBE cells	1.9 (24 h) ^g^	Expression	Tan *et al.* (2010) [95]
NQO1	SF	2 µM	*in vitro*; human NHBE cells	11.8 (6d) ^g^	Protein	Tan *et al.* (2010) [95]
NQO1	SF	9 µmol per day	*in vivo*; ICR mice; small intestine cells	1.6	Activity	Thimmulappa *et al.* (2002) [79]
NQO1	SF	9 µmol per day	*in vivo*; ICR mice; small intestine cells	2.5	Expression	Thimmulappa *et al.* (2002) [79]
NQO1	SF	50 µM	*in vitro*; human Caco-2 cells	2.5	Expression	Traka *et al.* (2005) [97]
NQO1	SF	100 nM	*in vitro*; mouse cortical neurons	8	Activity	Vauzour *et al.* (2010) [83]
NQO1	SF	4 µM	*in vitro*; mouse embryonic fibroblasts	2.5	Activity	Zhang *et al.* (2006) [91]
NQO1	SF	8 µM	*in vitro*; mouse embryonic fibroblasts	2.5	Activity	Zhang *et al.* (2006) [91]
NQO1	SF	4 µM	*in vitro*; rat bladder NBT-II cells	2.3	Activity	Zhang *et al.* (2006) [91]
NQO1	SF	8 µM	*in vitro*; rat bladder NBT-II cells	2.6	Activity	Zhang *et al.* (2006) [91]
NQO1	SF	1 µM	*in vitro*; TRAMP C1 cells	2.1	Expression	Zhang *et al.* (2013) [102]
NQO1	SF	2.5 µM	*in vitro*; TRAMP C1 cells	2.3	Expression	Zhang *et al.* (2013) [102]
SOD	I3C	10 µM	*in vitro*; HepG2 cells	1.3	Expression	Krajka-Kuźniak *et al.* (2015) [44]
SOD	I3C	10 µM	*in vitro*; HepG2 cells	1.2	Protein	Krajka-Kuźniak *et al.* (2015) [44]
SOD	I3C	2 µM	*in vitro*; HepG2 cells	1.2	Expression	Krajka-Kuźniak *et al.* (2015) [44]
SOD	I3C	2 µM	*in vitro*; HepG2 cells	1.2	Protein	Krajka-Kuźniak *et al.* (2015) [44]
SOD	PEITC	1 µM	*in vitro*; HepG2 cells	1.6	Expression	Krajka-Kuźniak *et al.* (2015) [44]
SOD	PEITC	1 µM	*in vitro*; HepG2 cells	1.2	Protein	Krajka-Kuźniak *et al.* (2015) [44]
SOD	PEITC	5 µM	*in vitro*; HepG2 cells	2.4	Expression	Krajka-Kuźniak *et al.* (2015) [44]
SOD	PEITC	5 µM	*in vitro*; HepG2 cells	1.4	Protein	Krajka-Kuźniak *et al.* (2015) [44]
TXNRD	Erucin	1 µM	*in vitro*; human MCF-7 cells	2.7 (8 h) ^g^	Expression	Wang *et al.* (2005) [103]
TXNRD	Erucin	12 µM	*in vitro*; human MCF-7 cells	7.3 (8 h) ^g^	Expression	Wang *et al.* (2005) [103]
TXNRD	Erucin	3 µM	*in vitro*; human MCF-7 cells	4.3 (8 h) ^g^	Expression	Wang *et al.* (2005) [103]
TXNRD	Erucin	6 µM	*in vitro*; human MCF-7 cells	5.6 (24 h) ^g^	Expression	Wang *et al.* (2005) [103]
TXNRD	Erucin	12 µM; 48 h after treatment	*in vitro*; human MCF-7 cells	4	Activity	Wang *et al.* (2005) [103]
TXNRD	Erucin	12 µM; 48 h after treatment	*in vitro*; human MCF-7 cells	4	Protein	Wang *et al.* (2005) [103]
TXNRD	Iberin	1 µM	*in vitro*; human MCF-7 cells	3.7 (8 h) ^g^	Expression	Wang *et al.* (2005) [103]
TXNRD	Iberin	3 µM	*in vitro*; human MCF-7 cells	4.4 (8 h) ^g^	Expression	Wang *et al.* (2005) [103]
TXNRD	Iberin	6 µM	*in vitro*; human MCF-7 cells	5.6 (24 h) ^g^	Expression	Wang *et al.* (2005) [103]
TXNRD	Iberin	12 µM	*in vitro*; human MCF-7 cells	5.8 (8 h) ^g^	Expression	Wang *et al.* (2005) [103]
TXNRD	Iberin	12 µM; 48 h after treatment	*in vitro*; human MCF-7 cells	4	Activity	Wang *et al.* (2005) [103]
TXNRD	Iberin	12 µM; 48 h after treatment	*in vitro*; human MCF-7 cells	3	Protein	Wang *et al.* (2005) [103]
TXNRD	SF	5 µM	*in vitro*; rat cardiomyocytes	2–2.7 ^d^	Activity	Angeloni *et al.* (2009) [82]
TXNRD	SF	5 µM	*in vitro*; rat cardiomyocytes	1.2–1.5 ^d^	Expression	Angeloni *et al.* (2009) [82]
TXNRD	SF	5 µM	*in vitro*; rat cardiomyocytes	1.5–1.9 ^d^	Protein	Angeloni *et al.* (2009) [82]
TXNRD	SF	10 µM	*in vitro*; human Caco-2 cells	2.2 (25 m) ^g^	Expression	Bacon *et al.* (2007) [104]
TXNRD	SF	10 µM	*in vitro*; human Caco-2 cells	1.7 (50 m) ^g^	Protein	Bacon *et al.* (2007) [104]
TXNRD	SF	2 µM; 24 h after treatment	*in vitro*; human Caco-2 cells	1.1	Expression	Bacon *et al.* (2007) [104]
TXNRD	SF	5 µM; 24 h after treatment	*in vitro*; human Caco-2 cells	1.7	Expression	Bacon *et al.* (2007) [104]
TXNRD	SF	10 µM; 24 h after treatment	*in vitro*; human Caco-2 cells	2.2	Expression	Bacon *et al.* (2007) [104]
TXNRD	SF	20 µM; 24 h after treatment	*in vitro*; human Caco-2 cells	3.5	Expression	Bacon *et al.* (2007) [104]
TXNRD	SF	2 µM; 48 h after treatment	*in vitro*; human Caco-2 cells	1.1	Protein	Bacon *et al.* (2007) [104]
TXNRD	SF	5 µM; 48 h after treatment	*in vitro*; human Caco-2 cells	1.4	Protein	Bacon *et al.* (2007) [104]
TXNRD	SF	10 µM; 48 h after treatment	*in vitro*; human Caco-2 cells	1.6	Protein	Bacon *et al.* (2007) [104]
TXNRD	SF	20 µM; 48 h after treatment	*in vitro*; human Caco-2 cells	1.5	Protein	Bacon *et al.* (2007) [104]
TXNRD	SF	10 µM	*in vitro*; human HepG2 cells	4 (25 m) ^g^	Expression	Bacon *et al.* (2007) [104]
TXNRD	SF	10 µM	*in vitro*; human HepG2 cells	2.2 (50 m) ^g^	Protein	Bacon *et al.* (2007) [104]
TXNRD	SF	2 µM; 24 h after treatment	*in vitro*; human HepG2 cells	2.1	Expression	Bacon *et al.* (2007) [104]
TXNRD	SF	5 µM; 24 h after treatment	*in vitro*; human HepG2 cells	2.7	Expression	Bacon *et al.* (2007) [104]
TXNRD	SF	10 µM; 24 h after treatment	*in vitro*; human HepG2 cells	2.5	Expression	Bacon *et al.* (2007) [104]
TXNRD	SF	20 µM; 24 h after treatment	*in vitro*; human HepG2 cells	0.8	Expression	Bacon *et al.* (2007) [104]
TXNRD	SF	2 µM; 48 h after treatment	*in vitro*; human HepG2 cells	1.5	Protein	Bacon *et al.* (2007) [104]
TXNRD	SF	5 µM; 48 h after treatment	*in vitro*; human HepG2 cells	2	Protein	Bacon *et al.* (2007) [104]
TXNRD	SF	10 µM; 48 h after treatment	*in vitro*; human HepG2 cells	2.7	Protein	Bacon *et al.* (2007) [104]
TXNRD	SF	20 µM; 48 h after treatment	*in vitro*; human HepG2 cells	2.1	Protein	Bacon *et al.* (2007) [104]
TXNRD	SF	102 µmol single dose	Human clinical; gastric mucosa	1.5	Expression	Gasper *et al.* (2007) [80]
TXNRD	SF	344 µmol single dose	Human clinical; gastric mucosa	2.1 (1.6) ^c^	Expression	Gasper *et al.* (2007) [80]
TXNRD	SF	50 mg per kg; 6 h after i.p. injection	*in vivo*; *tub/tub* P14 mice; retinal cells	2.4	Protein	Kong *et al.* (2007) [105]
TXNRD	SF	50 mg per kg; 12 h after i.p. injection	*in vivo*; *tub/tub* P14 mice; retinal cells	1.8	Protein	Kong *et al.* (2007) [105]
TXNRD	SF	50 µM	*in vitro*; human Caco-2 cells	8.8	Expression	Traka *et al.* (2005) [97]
TXNRD	SF	0.1 µM	*in vitro*; mouse cortical neurons	2.6	Activity	Vauzour *et al.* (2010) [83]
TXNRD	SF	1 µM	*in vitro*; human MCF-7 cells	3.4 (8 h) ^g^	Expression	Wang *et al.* (2005) [103]
TXNRD	SF	3 µM	*in vitro*; human MCF-7 cells	4.1 (8 h) ^g^	Expression	Wang *et al.* (2005) [103]
TXNRD	SF	6 µM	*in vitro*; human MCF-7 cells	4.8 (24 h) ^g^	Expression	Wang *et al.* (2005) [103]
TXNRD	SF	12 µM	*in vitro*; human MCF-7 cells	5.4 (8 h) ^g^	Expression	Wang *et al.* (2005) [103]
TXNRD	SF	12 µM; 48 h after treatment	*in vitro*; human MCF-7 cells	5	Activity	Wang *et al.* (2005) [103]
TXNRD	SF	12 µM; 48 h after treatment	*in vitro*; human MCF-7 cells	3	Protein	Wang *et al.* (2005) [103]
TXNRD1	SF	90 mg/kg; 3 h after treatment	*in vivo*; Nrf2(+/+) mice; liver	2.6	Expression	Hu *et al.* (2006) [81]
TXNRD1	SF	90 mg/kg; 12 h after treatment	*in vivo*; Nrf2(+/+) mice; liver	2	Expression	Hu *et al.* (2006) [81]
TXNRD3	SF	90 mg/kg; 3 h after treatment	*in vivo*; Nrf2(+/+) mice; liver	2.4	Expression	Hu *et al.* (2006) [81]
UGT family 2	SF	9 µmol per day	*in vivo*; ICR mice; small intestine cells	8	Expression	Thimmulappa *et al.* (2002) [79]
UGT1A1	SF	11 µM in onion/broccoli extract	Human clinical; enterocytes	2.4	Expression	Petri *et al.* (2003) [86]
UGT1A1	SF	11 µM in onion/broccoli extract	*in vitro*; human Caco-2 cells	1.5	Expression	Petri *et al.* (2003) [86]
UGT1A6	SF	9 µmol per day	*in vivo*; ICR mice; small intestine cells	1.4	Expression	Thimmulappa *et al.* (2002) [79]

^a^ Fold changes were at times estimated from graphs or approximated from reported ranges; ^b^ Treatments involved a mixture of glucosinolate hydrolysis products reported in more detail in the corresponding reference; ^c^ The first numeral indicates fold change based on qRT-PCR experiments. Values in parentheses indicate mean fold changes for all reported microarray probes of a given gene; ^d^ Range of significant fold changes over a time course; ^e^ Isoform not given; ^f^ Range of fold changes for several GST isoforms quantified using a microarray; ^g^ Values reported are the most significant positive fold change following treatment with the indicated compound or plant extract for different time periods. The treatment period is indicated in parentheses following the fold change; ^h^ Range reported in text; numerical values from Western blots not reported in tables or figures; ^i^ Values are mean fold changes from four prostate cancer cell lines: LNCaP, MDA Pca 2a, MDA Pca 2b, and TSU-Pr1; *Abbreviations:* ARPE, arising retinal pigment epithelial; BITC, benzyl isothiocyanate; CAT, catalase; DIM, 3,3′-diindolylmethane; GCL, glutamate-cysteine ligase; GCLC, glutamate-cysteine ligase catalytic subunit; GPX, glutathione peroxidase; GSH, glutathione; GSR, glutathione reductase; GST, glutathione S-transferase (A, alpha; P, pi; M, mu; T, tau); GSHP, glucosinolate hydrolysis product; HO-1, heme oxygenase 1; I3C, indole-3-carbinol; ITC, isothiocyanate; NHBEC, normal human bronchial epithelial cells; NQO1, NADPH:quinone oxidoreductase-1; PEITC, phenethyl isothiocyanate; SF, sulforaphane; TXNRD, thioredoxin reductase; UGT, UDP-glucuronosyltransferase.
